# Revealing Catalytic Properties of Palladium/Gold Systems toward Hydrogen Evolution, Oxidation, and Absorption with Scanning Electrochemical Microscopy

**DOI:** 10.1021/acscatal.5c00783

**Published:** 2025-05-14

**Authors:** Christian M. Schott, Julia Holl, Raul Zazpe, Michael Kopp, Ondřej Man, Sitaramanjaneya M. Thalluri, Jhonatan Rodriguez-Pereira, Peter M. Schneider, Kun-Ting Song, Emre Keles, Pekka Peljo, Jerzy J. Jasielec, Elena L. Gubanova, Jan M. Macak, Aliaksandr S. Bandarenka

**Affiliations:** 1 Physics of Energy Conversion and Storage, 84665 Technical University of Munich, James Franck Str. 1, Garching 85748, Germany; 2 Central European Institute of Technology, 48274 Brno University of Technology, Purkynova 123, Brno 61200, Czech Republic; 3 Center of Materials and Nanotechnologies, Faculty of Chemical Technology, 48252 University of Pardubice, Nam. Cs. Legii 565, Pardubice 53002, Czech Republic; 4 Research Group of Battery Materials and Technologies, Department of Mechanical and Materials Engineering, Faculty of Technology, 8058 University of Turku, Turun, Yliopisto 20014, Finland; 5 Department of Physical Chemistry and Modelling, Faculty of Materials Science and Ceramics, AGH University of Science and Technology, Al. Mickiewicza 30, Kraków 30-059, Poland; 6 Catalysis Research Center TUM, Ernst-Otto-Fischer-Str. 1, Garching 85748, Germany

**Keywords:** scanning electrochemical microscopy, hydrogen oxidation reaction, hydrogen evolution reaction, hydride formation, monolayer, nanostructures, palladium

## Abstract

Palladium (Pd) is an active catalyst for various reactions, such as hydrogen evolution (HER) and hydrogen oxidation (HOR) reactions. However, its activity can be further optimized by introducing strain and ligand effects from Pd deposition onto suitable substrates like gold (Au). In this study, we use scanning electrochemical microscopy (SECM) to investigate the catalytic properties of such Pd/Au systems. For the HER, a sub-monolayer of Pd (Pd_ML_) was electrochemically deposited onto half of a polycrystalline (pc) Au substrate with underpotential deposition (UPD). The localized activity measurements revealed improved HER kinetics for Pd atoms at the Pd/Au border in 0.1 M HClO_4_. As a consequence, a set of Pd/Au samples with increasing density of Pd/Au borders was synthesized by atomic layer deposition (ALD). These ALD Pd deposits have an increased thickness compared to a sub-monolayer, which makes hydride formation thermodynamically viable. Because of this, the samples were investigated for the HOR/H absorption activity using the redox competition (RC) mode. We highlight the influence of cations in 0.1 M AMOH (AM = Li^+^, Na^+^, K^+^, Rb^+^, Cs^+^) electrolytes on the HOR/H absorption activity, displaying higher activities for larger cations: j_LiOH_ < j_NaOH_ < j_KOH_ < j_RbOH_ < j_CsOH_. From the spatial and temporal resolution of the activity, active spots are identified, which expand with time and diminishing hydrogen concentration in the electrolyte. Additional laser-induced current transient (LICT) experiments confirm the dependency between cation and electrocatalytic activity observed with RC-SECM.

## Graphical abstract

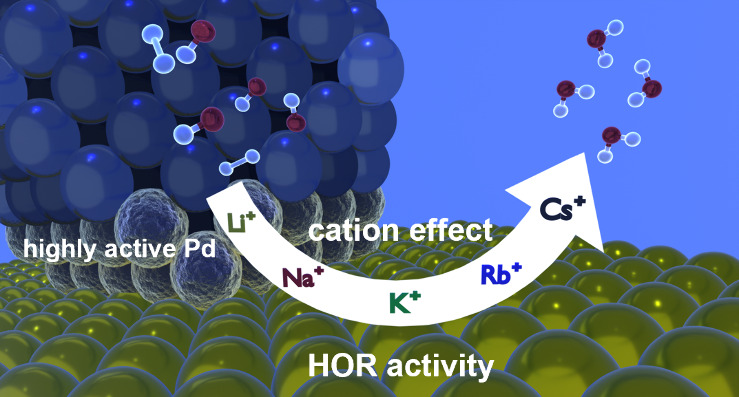

## Introduction

Palladium (Pd) is a promising catalyst for the hydrogen evolution reaction (HER)^1,2^ and hydrogen oxidation reaction (HOR),^3,4,5^ due to its almost ideal, slightly negative adsorption energy of atomic hydrogen.^6,7^ As a result, the desorption of the reaction products limits the rate of the HER/HOR.^7,8^ Many strategies exist to modify the adsorption energy, for instance, by varying the crystal orientation, surface roughness, or support materials.^7,9^ Furthermore, the formation of bulk alloys seems to influence the binding energies through strain and ligand effects, whereas strain effects primarily shift adsorption energies for thin films thicker than three atomic layers in the absence of ligand effects.^10^ These observations have led to studies of thin Pd films deposited on single-crystal substrates^11,12^ which alter the hydrogen adsorption energy by shifting the d-band center of Pd.^13^ For example, Liang et al.^14^ employed noise electrochemical scanning tunneling microscopy (n-EC-STM) experiments to reveal that the most active sites are populated at the boundary between two-dimensional Pd clusters and the Au substrate. Their work suggested maximizing the number of such two-dimensional Pd clusters to achieve the highest reaction activity for the HER.

Despite the benefits toward HER/HOR activity of Pd/Au catalytic systems, they face stability problems, such as alloying at room temperature^15,16^ or rapid dissolution during electrochemical oxidation-reduction cycles.^17^ Degradation was also reported for a Pd/Au­(pc) surface with a coverage of 0.39, underlining a decrease in electrochemically active surface area and, therefore, electrocatalytic activity after three cathodic scans of HER/HOR in H_2_-saturated 0.1 M NaOH.^15^ Besides alkaline media, instability was observed in 0.1 M H_2_SO_4_, where a 0.8 mono layer (ML) of Pd deposited on Au(111) displayed changes in cyclic voltammetry measurements, arguably forming a uniform layer through the merging of Pd islands.^18^ Furthermore, we want to highlight the property of Pd to form hydrides, also known as the absorption (abs) of atomic hydrogen into the Pd crystal structure. Hydride formation occurs for potentials below ∼0.3 V vs. the reversible hydrogen electrode (RHE) in 0.1 M HClO_4_ for Pd/C nanostructures.^19^ For Pd deposits on Au, H_abs_ takes place for film thicknesses larger than 2 ML.^18,20^ Nevertheless, the hydrogen to Pd ratio becomes larger for more cathodic potentials,^19^ and the threshold potential at which hydrogen absorbs into the crystal most likely depends on the electrolyte and the Pd film thickness. Hydride formation leads to the development of defects at the nanometer level due to the interaction between hydrogen and Pd atoms during the diffusion process.^21^ These surface reconstructions have been investigated by STM in ultra-high vacuum (UHV)^22^ and under electrochemical conditions.^23^ The high-resolution images in UHV conditions of Pd(110) reveal (1 × 2) missing-row structures originating from upper and lower terraces.^24^ EC-STM measurements for low-indexed Pd single crystals in 0.1 M HClO_4_ illustrate formations of hills, islands, and other hydride-induced morphological defects.^33^ It should be highlighted that, especially for thicker layers or deposits, reconstruction will be more dominant since hydrides will be formed until the size- ^25,26^ and potential-dependent^19^ H/Pd ratio is established.

Consequently, it is of major interest to record the localized activity on Pd/Au samples to visualize heterogeneities, such as those emerging from surface segregations, Pd deposit agglomerations, and hydride-induced defects. For more complex systems, such as Pd nanopillars on Au­(pc), localized measurements also provide information about the distribution of deposits on a substrate and, hence, reveal positions containing large agglomerates. Such localized activity measurements can be conducted by scanning electrochemical microscopy (SECM), which employs micro-/nanoelectrodes to locally reduce/oxidize free-diffusing species.^27^ Besides spatial activity assessment, it is crucial to specifically adapt the method to acquire temporal information as well. This combination of spatial and temporal evaluations identifies active areas and their evolution and progression during the operando reaction conditions.^28^

In this work, we employ SECM to investigate such Pd/Au systems for the HER and HOR reactions. For exploring the HER, a Pd sub-monolayer (Pd_ML_) was electrochemically deposited on one half of a polycrystalline (pc) Au sample to study the HER activity using the sample generation tip collection (SG-TC) mode.^27,29–32^ In this mode, the Pd/Au substrate is polarized to evolve molecular hydrogen, while the HOR takes place at the microelectrode. The larger the measured HOR current at the microelectrode tip, the greater the local HER activity of the catalyst sample.

Furthermore, Pd nanoclusters with different lateral sizes, thicknesses, and loadings were deposited on Au­(pc) using Atomic Layer Deposition (ALD). Since the thickness of the Pd clusters exceeds several monolayers of Pd, significant hydride formation will be thermodynamically feasible under HER conditions. As discussed above, hydride formation induces degradation of the (sub)­surface Pd layer due to induced surface reconstructions under reaction conditions.^33^ To minimize such structural modification, we investigated Pd nanopillar deposits on Au­(pc) for the HOR since, at those potentials, the extent of hydride formation will be less significant, as highlighted in ref 19.

While HOR measurements are often performed via tip-generation sample collection (TG-SC) mode,^34–37^ significant background currents on larger conductive substrates typically complicate visualizing electrocatalytic regions with varying activity.^38,39^ To avoid these limitations, the redox competition mode (RC-SECM) was developed in 2006 and is now considered the state-of-the-art method for investigating the local activity and selectivity of the oxygen reduction reaction (ORR) toward H_2_O or H_2_O_2_.^38,40,41^ To the best of our knowledge, we employ the RC-SECM mode for the first time to investigate the HOR. Besides localized information, the mode, most likely, minimizes hydride-induced reconstructions of Pd compared to macroscopic, more traditional measurements. The reason stems from the fact that the electrolyte is only locally purged with molecular hydrogen for short periods, which leads to smaller current densities and, therefore, less hydride formation. The mode allows for the elucidation of the cation influence on the HOR activity in 0.1 M AMOH (AM = Li^+^, Na^+^, K^+^, Rb^+^, Cs^+^) for Pd/Au systems, which has not been reported before in the literature with traditional measurements. Besides the localized activity assessment, an evaluation method for the RC-SECM mode was developed that provides a more “macroscopic” parameter to quantify the HOR/H_abs_, similar to more traditional methods. To validate the RC-SECM results and to prove the observed cation influence on the HOR activity, laser-induced current transient technique (LICT) measurements were performed on electrochemically deposited sub-Pd_ML_ on Au­(pc) and Au(111). The LICT technique allows for the estimation of the so-called potential of maximum entropy (PME), a parameter related to the structure of the electrochemical double layer formed at the catalyst material under defined conditions. Studies showed that the closer the PME is positioned to the thermodynamic equilibrium potential of the electrocatalytic reaction, the more active this reaction seems to be.^42–44^ Our LICT experiments predict the same cation trend on the electrocatalytic activity as our developed RC-SECM, suggesting an increased HOR activity with increasing cation size (Cs^+^> K^+^> Na^+^> Li^+^). The observed trend is in contrast to the HER trend reported by Bender et al.,^45^ detected for a bulk Pd­(pc) disc, where the highest activities were obtained with decreasing cation size (Li^+^> Na^+^> K^+^> Cs^+^). The different trend indicates the crucial role of Pd atoms in the vicinity of the Pd/Au border,^14^ whose d-band structure is shifted due to strain and ligand effects from the underlying Au­(pc) substrate.

## Method Section

### 2.1 Microelectrode Fabrication and Characterization

One end of a glass capillary with a diameter of 1.5 mm was mechanically pulled into a tip, in which a piece of Pt wire (25 μm diameter, 99.95% metals basis, Alfa Aesar) was inserted and subsequently sealed. The end of the Pt wire inside the capillary was soldered to a Cu wire (0.5 mm diameter, grade 2, SchneiTec) using Sn_96.5_Ag_3_Cu_0.5_ solder (0.5 mm diameter, Felder Löttechnik). The tip of the microelectrode was polished with three different Al_2_O_3_ pastes (5 μm, 1 μm, and 0.3 μm) and silicon dioxide particles of 0.03 μm size (Sensolytics, Germany). After each polishing step, the tip was rinsed multiple times with deionized water (18.2 MΩ cm, Stakpure, Germany) and ethanol (absolute for analysis, EMSURE ACS) and was cleaned in an ultrasonic bath (Bandelin). Microelectrodes with glass-to-Pt ratios between 6 to 10 were electrochemically characterized in a redox mediator containing 5 mM potassium hexacyanoferrate­(II) trihydrate K_4_[Fe­(CN)_6_] · 3H_2_O (99.95% trace metals basis, Sigma-Aldrich) and 100 mM potassium chloride KCl (99.99% trace metals basis, Sigma-Aldrich). Here, cyclic voltammetry (CV) was performed using a bipotentiostat from Autolab (PGSTAT302N) in the range from −0.2 to 0.5 V vs. a silver/silver chloride (Ag/AgCl) reference electrode (Metrohm, Germany). The diffusion-limited Fe­(CN)_6_^4–^ oxidation current was compared to the theoretical value of 15.6 nA. Under experimental conditions, slightly higher currents emerge, highlighting an increased surface area. Only microelectrodes with similar diffusion-limited currents were selected to ensure consistent electrocatalytic behavior in subsequent SECM experiments. A more detailed description can be found in the ref 46.

### 2.2 SECM Setup

The microelectrode characterization in a KCl electrolyte with K_4_[Fe­(CN)_6_] 3H_2_O was performed in an acrylic glass cell (Sensolytics, Germany). Electrochemical measurements involving HClO_4_, or AMOH (AM = Li^+^, Na^+^, K^+^, Rb^+^, Cs^+^) were conducted in a PEEK cell. In the acrylic glass cell, a curled miniature Pt counter electrode (Sensolytics, Germany) was employed, while the PEEK cell utilized a Pt net counter electrode. Both cells used an Ag/AgCl reference electrode containing a 3 M KCl filling electrolyte. The microelectrode was attached to the SECM stepper stage, permitting movement in all three dimensions. The setup was configured as either a three-electrode or four-electrode system, depending on the specific electrochemical experiment.

### 2.3 Electrolytes

Before any experiments, the SECM cells were washed multiple times with boiled, deionized water. Measurements in different electrolytes for the Pd/Au samples were conducted above the same sample area. Between measurements, the previous electrolyte was removed, and the cell was flushed multiple times with deionized water before the next electrolyte was filled into the cell. In the PEEK cell, six different electrolytes were used: 0.1 M HClO_4_ (70% HClO_4_, extra pure, Acros) and 0.1 M AMOH (AM = Li^+^, Na^+^, K^+^, Rb^+^, Cs^+^). For the hydroxide electrolytes, 100 ml solutions were prepared by dissolving approximately 0.24 g of LiOH powder (anhydrous, 99.995% metals basis, Alfa Aesar), 0.4 g of NaOH pellets (semiconductor grade, 99.99% trace metals basis, Sigma-Aldrich), and 0.56 g of KOH pellets (semiconductor grade, 99.99% trace metals basis, Sigma-Aldrich), respectively, in deionized water. For RbOH and CsOH, solutions with 50 wt % in H_2_O and 99% trace metals basis (Sigma-Aldrich) were used, with volumes of 1.178 ml and 1.743 ml, respectively. Similar electrolytes were used for the LICT experiments.

### 2.4 Underpotential Deposition of a Pd Sub-monolayer onto an Au Substrate

The underpotential deposition (UPD) of a Pd sub-monolayer was performed in a glass cell, which was previously cleaned using Caro’s Acid, a 3:1 mixture of sulfuric acid (96% H_2_SO_4_, Suprapur, Merck, Germany) and hydrogen peroxide (30% H_2_O_2_, p.a., ISO, Carl Roth, Germany). After the acid remained for ∼12 h in the cell, all glass parts were washed multiple times with boiled deionized water. The counter and reference electrode during the UPD corresponded to a curled Pd wire and mercury/mercurous sulfate reference electrode (Si Analytics, Germany), respectively. The deposition procedure was conducted according to ref 47, where linear sweep voltammetry (LSV) was executed in an Ar-saturated electrolyte of 0.1 M H_2_SO_4_ and 0.1 mM H_2_PdCl_4_ on a clean Au substrate. For the LSV, a potential range from 0.94 to 0.26 V vs. RHE was used with a scan rate of 1 mV s^–1^. In our work, two Au substrates were employed, corresponding to either Au­(pc) (QCM chip, Stanford Research Systems) or Au(111) (diameter: 10 mm, 99.999%, MaTecK, Jülich, Germany). Before UPD, the Au(111) single crystal was annealed in a tubular furnace (Heraeus Instruments RO 7/50) in an Ar atmosphere at 450 °C for 20 min. To validate the successful annealing procedure, CVs with a potential range from 0.52 to 1.72 V vs. RHE were recorded in Ar-saturated (99.999% purity, Air Liquide) 0.1 M HClO_4_ with a scan rate of 50 mV s^–1^.

### 2.5 Atomic Layer Deposition of Pd Films on Au­(pc)

Au­(pc) substrates were cleaned before ALD processes by dipping them in an ultrasonic bath in acetone, isopropanol, and distilled water for 5 min at each and dried with N_2_ flow. Pd was deposited onto Au­(pc) using an ALD tool (thermal ALD, TFS 200, Beneq). Palladium­(II) hexafluoroacetylacetonate (min. 95%, Strem) and formaldehyde (36-38%) were used as the precursor and co-reactant, respectively. Under these deposition conditions, one ALD cycle was defined by the following sequence: Pd pulse (500 ms) - exposure time (10 s) - N_2_ purge (20 s) - formaldehyde pulse (1 s) - exposure time (10 s) - N_2_ purge (20 s). The number of ALD cycles applied was 600, 1000, and 1600, respectively (to induce the formation of Pd nanopillars of different sizes and coverage). All processes were carried out at a temperature of 200 °C, using N_2_ (99.9999%) as the carrier gas at a flow rate of 400 sccm.

### 2.6 Materials Characterization

Scanning electron microscope (SEM) analyses were carried out by field-emission SEM JEOL JSM 7500F operated at 5 kV using an in-lens detector with the combined secondary electron as well as backscattered electron imaging contributions. Dimensions of Pd nanopillars were obtained by the statistical analyses of SEM images using proprietary Nanomeasure software and using at least 30 counts for each sample (n ≥ 30).

Electron Back Scatter Diffraction (EBSD) analyses were conducted using an SEM-FIB system FEI Helios 660 G3 at 25 kV accelerating voltage and 1.6 nA beam current. The original analyzed area was 8 μm x 16 μm with a 10 nm step. The collected diffraction data had to be cropped to 8 μm × ∼10 μm to compensate the effect of drift. EBSD data were treated in OIM Analysis 8 (EDAX) software by reindexing and pattern reconstruction to mitigate the effect of the very fine size of the Au/Pd particles.

The roughness and morphology of Pd nanopillars were determined by atomic force microscopy (AFM) in air using the NTEGRA (NT-MDT) system and applying tapping mode with a HA-HR tip (ScenSans) and a step of 8 nm. The roughness value was obtained as the mean value of 3 measurements of a scanned area of 1 × 1 μm2.

To verify the surface chemical composition of the Pd deposited by ALD, X-ray photoelectron spectroscopy (XPS) analysis was performed using Scienta-Omicron (ESCA-2SR) instrument, with Al–Kα monochromatic X-ray source (*h*ν = 1486.69 eV). The acquired spectra were further fitted using CasaXPS software and referenced to the Au^0^ species centered at 84.0 eV, in order to perform the binding energy scale correction. Pd 3d spectra were fitted using a Shirley-type background with asymmetric Lorentzian function LA (1.10, 1.8, 120) for the metallic state (Pd^0^) and mixed Gaussian-Lorentzian functions GL (30) for Pd oxides and Pd plasmon loss.

### 2.7 SECM Experimental Procedure

Before approaching the sample with the microelectrode tip, the microelectrode was cleaned and electrochemically activated by executing CVs in 0.1 M HClO_4_, with a potential range from −0.02 V to 1.3 V vs. RHE at a scan rate of 0.1 V s^–1^, and potential steps of 0.01 V. The microelectrode was first manually approached and then further positioned using the stepper motor of the SECM device. Approximately 0.47 V vs. RHE was applied to the microelectrode in 0.1 M HClO_4_ under air-saturated conditions. At this potential, the ORR occurs at the tip of the microelectrode. By approaching the microelectrode at a speed of 2 μm s^–1^ and in increments of 2 μm, a continuous decrease in the ORR current was observed due to the blocked oxygen diffusion to the microelectrode as the tip-to-sample distance decreased. When the current dropped below 1 nA, the approach increment was reduced to 1 μm with a speed of 1 μm s^–1^. The low increments and approaching speeds were chosen to ensure that the tip is not destroyed by slightly touching the surface of the sample, indicated by a sudden large, recorded current. The microelectrode was then retracted by 10 μm to establish a fixed and well-known tip-to-sample distance. The samples were cleaned and activated in 0.1 M HClO_4_ before the SECM experiments. The potential ranges in the respective CVs corresponded to 0.1 V to 0.6 V vs. RHE with a scan rate of 0.1 V s^–1^ and potential steps of 0.01 V. For all array scan measurements, an increment of 12.5 μm and a maximum speed of 12.5 μm s^–1^ were used. The waiting time between measurements corresponds to 25 ms during the SG-TC experiments and 100 ms for the RC-SECM measurements. For the SG-TC experiments, the microelectrode potential was set to 0.04 V vs. RHE, while the sample potential was set to 0.015 V vs. RHE. Due to air-saturated electrolyte conditions, the equilibrium potential of the HER/HOR was slightly Nernstian shifted, resulting in the production of molecular hydrogen at slightly positive potentials versus the RHE scale. Different potential pulses were used for the redox competition (RC) experiments, and their purposes are discussed in the results section. The RC experiments for different electrolytes were conducted over the same area of the respective Pd nanopillar sample. During the exchange procedure of the electrolytes, the position of the microelectrode was maintained, allowing measurements of the same area of the sample across different electrolytes to ensure ideal conditions for activity comparison. For completeness, it should be noted that for experiments with the Pd nanopillar sample after 1000 ALD cycles, a tilt correction was employed using the piezo scanning stage of the SECM.

### 2.8 Laser-Induced Current Transient Technique

LICT experiments were performed in a glass cell consisting of a preconditioning cell and a main compartment. Prior to any measurements, the entire cell was washed multiple times with boiled deionized water, followed by cold water. For the counter electrode, a Pt wire was employed, while the reference electrode corresponded to a mercury/mercurous sulfate electrode. The LICT experiments were conducted by recording the current at each respective potential during the pulsing of the laser beam (Δ*t* = 8 ns, *f* = 10 Hz, *P* = 150 mW, Quanta-Ray INDI Pulsed Nd:YAG laser, Spectra-Physics, USA) for 4 s. The potential was varied in a range from 0.53 to 0.71 V vs. RHE in 0.02 V steps for 0.1 M LiOH, NaOH, and KOH. The range was modified for RbOH to 0.47 V to 0.71 V and CsOH to 0.43 V to 0.67 V vs. RHE. The measurements were carried out three times in a positive/negative direction to ensure reproducibility.

To correct for background noise, the median value was subtracted from the recorded current transients. Selected peaks were then integrated over time to obtain the charge associated with interfacial water reorientation and specific adsorption. The charge values for the respective potentials recorded during the LICT measurements were plotted against the potentials. The intersection points with the *x*-axis, determined from linear fits through all measurement points, estimated the PME for the working electrode in the measured electrolyte.

## Results and Discussion

A sub-monolayer of Pd was deposited onto half of an Au­(pc) substrate via UPD^47^ to explore the local HER activity differences at the Pd/Au border. The successful Pd deposition on Au was previously confirmed by CV, as shown in Figure 1a**,** which reveals the characteristic features of Pd in 0.1 M HClO_4_.^2^ The study was motivated by the results from Liang et al.,^14^ who reported an activity increase of Pd atoms in the proximity of Pd/Au boundaries using n-EC-STM. The method identifies active positions by analyzing the noise emerging in the tunneling current under reaction conditions.^48^ The n-EC-STM differs from other electrochemical procedures, such as SECM, where the active areas are assessed by distinct faradaic currents recorded from the local reduction and/or oxidation processes of reactions.^27^ For the HER, the SG-TC mode (Figure 1b) of the SECM is employed to generate hydrogen at the sample and locally oxidize it at the microelectrode. Figure 1c displays the HOR microelectrode current in two- and three-dimensional maps at the Pd/Au border in air-saturated 0.1 M HClO_4_. The oxidation current indirectly estimates the spatial HER activity, depending on the amount of locally generated hydrogen. The highest electrocatalytic HER activity appears within the first ∼200 μm adjacent to the Pd/Au border, evident by the larger HOR microelectrode current. Nevertheless, the activity diminishes with prolonged distance from the border, in agreement with the previous results from Liang et al.^14^ The greater extension of the HER activity up to ∼200 μm from the border might be explained by the usage of 25 μm microelectrodes at a working distance of 10 μm. The produced hydrogen diffuses through the electrolyte and influences the HOR current at other measurement positions, which is also known as broadening. We also want to highlight that the Pd deposited layer displays several surface defects, as evident from two circular areas marked by the green arrow.

**Figure 1:**
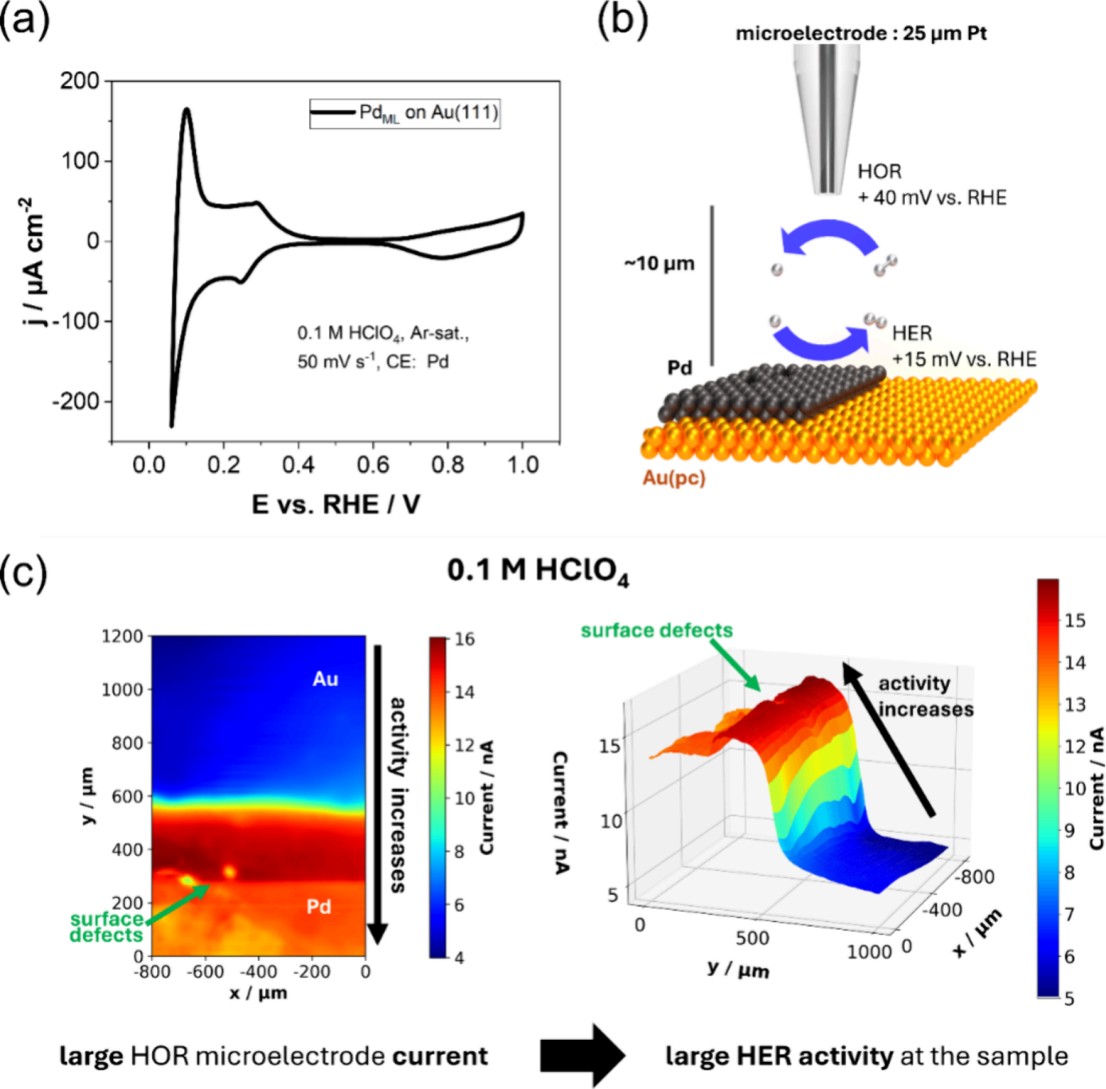
(a) Typical CV of the Pd_ML_ deposited on Au(111), recorded in Ar-saturated 0.1 M HClO_4_ at a scan rate of 50 mV s^–1^. (b) Schematic representation of the employed SG-TC mode at the interface between the deposited Pd and Au­(pc) used to study HER activities in air-saturated 0.1 M HClO_4_. Air-saturated electrolytes lead to a Nernstian potential shift, allowing for a hydrogen evolution reaction onset at an already slightly positive potential vs RHE. (c) Two- and three-dimensional HOR microelectrode current maps revealing the activity difference between the Pd and Au regions, as well as surface defects.

Samples with multiple active Pd/Au borders were synthesized using ALD. Pd nanopillars were grown onto an Au­(pc) substrate, as depicted in Figure 2a. The Pd nanopillars’ diameter ranges from 14 ± 3 nm to 21 ± 4 nm and 26 ± 6 nm, depending on the number of applied ALD cycles −600, 1000, and 1600 cycles, respectively. Figure S1 displays the XPS spectra for the Pd nanopillars on Au with 600 and 1000 cycles, suggesting an increase in the atomic concentration of Pd from ∼25% to ∼45%. The Pd appears to be mainly metallic since the binding energy of the spin-orbit splitting Pd 3d_5/2_ and Pd 3d_3/2_ is centered at ∼335.3 and 340.6 eV,^49^ respectively. A summary of the elemental atomic concentrations and Pd compositions is provided in Tables S1 and S2.

**Figure 2:**
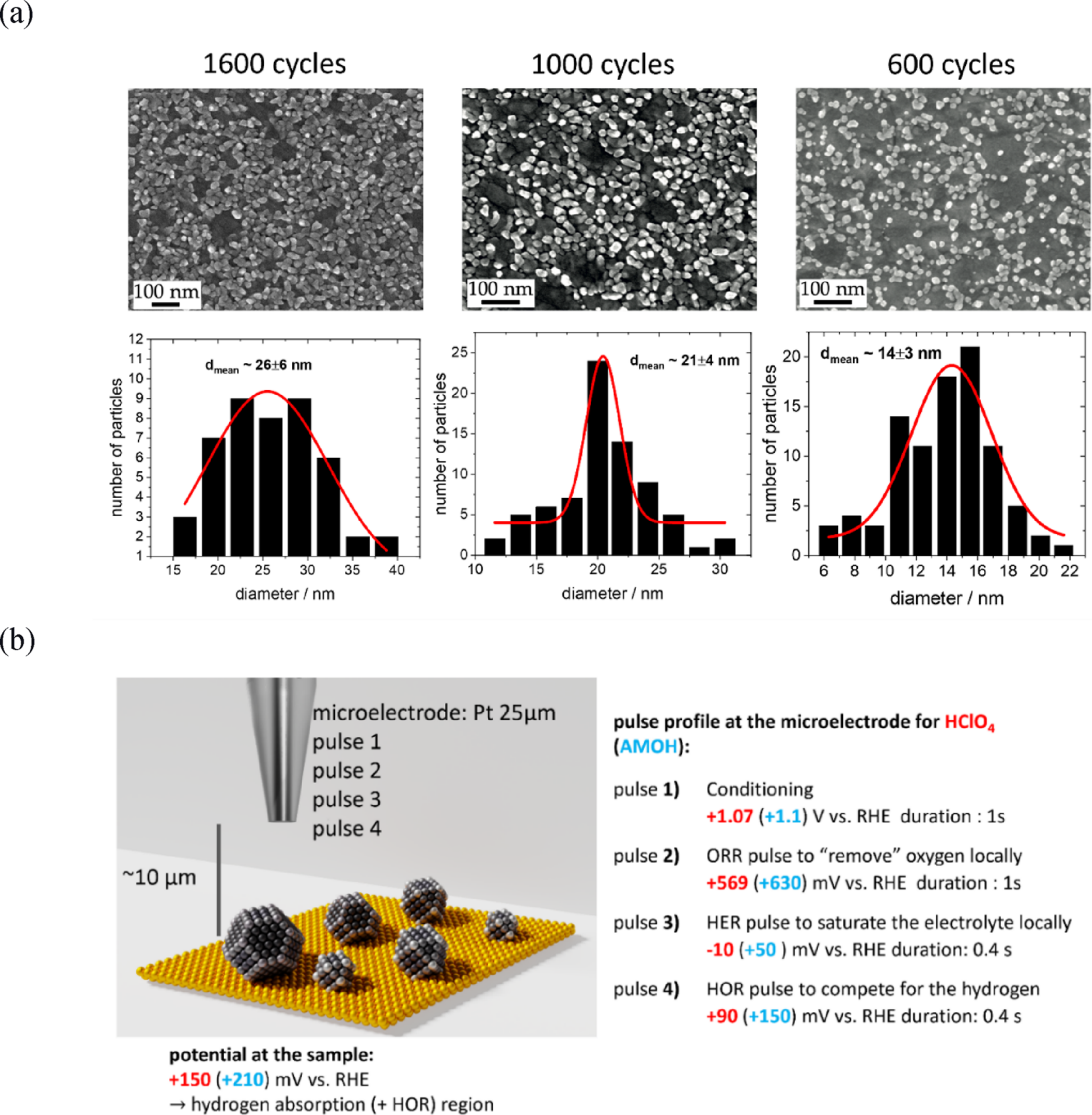
(a) SEM images of Pd nanopillars grown on Au­(pc) via ALD. Increased number of ALD cycles result in larger nanopillar sizes and higher Pd concentrations. (b) Schematic of the RC-SECM mode employed, illustrating the pulse profile at the microelectrode. Potentials for each pulse/sample bias in acidic and alkaline media are shown in red and blue, respectively.

These ALD Pd deposits with increased thickness - compared to a sub-monolayer - were investigated toward the HOR to minimize hydride formation^19^ and reconstructions of the Pd. The activity evaluations were performed with the RC-SECM mode, which is schematized in Figure 2b. A detailed description of the pulse in the mode can be found in the Supporting Information. A big advantage of RC-SECM in contrast to macroscopic measurements appears from the local H_2_ purging (pulse 3) of the electrolyte between microelectrode and sample instead of saturating the entire volume. Here, only minor H_2_ reactants are introduced for 0.4 s, resulting in fewer Pd–H interactions and smaller HOR currents compared to macroscopic experiments. Consequently, less hydrogen can diffuse into the Pd crystal structure, reducing the migration of Pd atoms through reconstruction. The local HOR/H_abs_ sample activity is acquired within the fourth pulse, at which the previously generated H_2_ is simultaneously oxidized at the microelectrode and the local sample position. A larger recorded microelectrode oxidation current suggests a less active sample position and vice versa. We can use the magnitude of the recorded HOR current to indirectly reveal the local sample activity. From the RC-SECM data, we created 100x150 μm^2^ HOR/H_abs_ activity maps of the sample for each millisecond, as comprehensively discussed in the Supporting Information. Since the H_2_ reactants are only introduced once during the third pulse, continuously diminishing during the fourth pulse, the method also visualizes the local change of the activity associated with a decrease in H_2_ concentration during the HOR.

Figure 3 displays activity maps for the Pd nanopillars with ∼26 ± 6 nm diameter supported on Au­(pc) for the first 4 ms in 0.1 M HClO_4_ and 0.1 M LiOH. We refer to the Supporting Information for detailed explanations (Figures S2 and S3) and activity maps in all electrolytes (Figures S4–S9), such as animated videos in Figure S10. Small values in the activity maps, indicated in blue, suggest larger HOR/H_abs_ electrocatalytic activities of the Pd/Au samples. At 0 ms, the most active spots in 0.1 M LiOH are detected at different positions compared to those in 0.1 M HClO_4_. Only the active region at *x* ∼ 100–120 μm and *y* ∼ −70 to −90 μm appears for both electrolytes. The differences might arise from reconstruction during the measurement conditions or from distinct HOR/H_abs_ mechanisms in acidic and alkaline media on Pd/Au. After 1–2 ms in acidic media, additional active spots appear on the activity maps while persisting spots expand or merge. In addition, all maps become darker (green and blue) with time, which is caused by the rapid oxidation of the previously introduced H_2_ reactants. Initially, a strong contrast was visible between more active and less active regions; however, as hydrogen was consumed, the electrocatalytic activity of distinct regions appeared to be more uniform. Similar observations were also found for measurements in 0.1 M LiOH and other hydroxides measured at the same sample area. The results also highlight that the initially induced H_2_ concentration decreases more slowly in 0.1 M LiOH than in 0.1 M HClO_4_, as suggested by the slower decrease in the background current over time. This aligns with the slower reaction kinetics for the HOR in alkaline media for Pd.^50^

**Figure 3:**
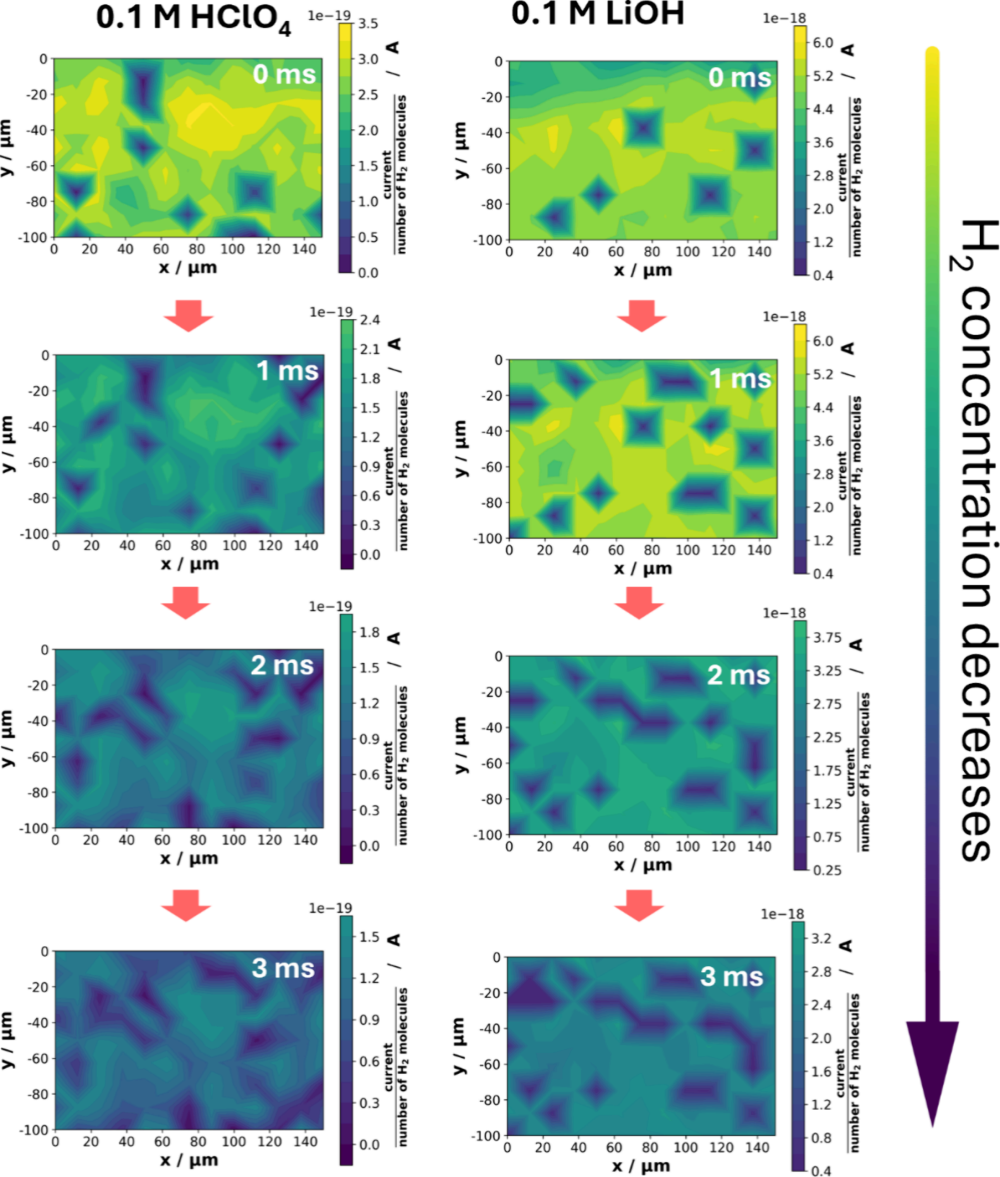
RC-SECM activity maps for the Pd nanopillar sample grown on Au­(pc) with an average diameter of 26 ± 6 nm. The heat maps display the HOR microelectrode current, normalized by the number of hydrogen molecules produced during the third pulse of RC-SECM. The plots on the left represent measurements in 0.1 M HClO_4_, while those on the right show corresponding measurements in 0.1 M LiOH at identical sample locations. The time in the top right corner of each plot indicates the specific moment during the fourth pulse when the HOR current was recorded. The arrow on the right signifies that with increasing time, the previously induced H_2_ concentration during the third pulse decreases, as evidenced by the color change in the heatmaps.

Besides the spatial resolution of the electrochemical activity, the RC-SECM also allows for the quantification of the activity map, providing a more “macroscopic” parameter for activity comparison at different electrolytes. The quantification was conducted through surface integration over the lateral coordinates for each activity map at each millisecond. A smaller value, obtained from the surface integral, corresponds to a higher electrocatalytic HOR/H_abs_ activity since a smaller HOR microelectrode current is recorded for a more active spot during the RC-SECM measurements. The method was validated in the Supporting Information by examining the HOR/H_abs_ activity of the three ALD Pd samples in 0.1 M HClO_4_, which contain distinct nanopillar sizes and content (Figure S11). Here, we will focus on the average HOR/H_abs_ activity dependency in 0.1 M AMOH (AM = Li^+^, Na^+^, K^+^, Rb^+^, Cs^+^). Figure 4 depicts the activity measurements in 0.1 M AMOH for each cation across the three Pd nanopillar samples of distinct sizes. For the 14 ± 3 nm- and 21 ± 4 nm-sized Pd nanopillars, the HOR/H_abs_ activity consistently increases with increasing cation size, and therefore, decreasing hydration energy j_LiOH_ < j_NaOH_ < j_KOH_ < j_RbOH_ < j_CsOH_. For the largest Pd nanopillars of 26 ± 6 nm, a similar tendency is observed; however, the HOR/H_abs_ activity in NaOH slightly exceeds that in KOH, i.e., j_LiOH_ < j_KOH_ < j_NaOH_ < j_RbOH_ < j_CsOH_. The question arises as to why the decreasing hydration energy of the cation correlates with an increase in the electrocatalytic HOR/H_abs_ activity of the samples. A recent study demonstrated that the HER activity of Pd increases with decreasing cation size and increasing hydration energy in 0.1 M AMOH electrolytes.^45^ These findings are in strong contrast with the trend reported for Au, where increased HER activity is associated with larger cation size and smaller hydration energy.^51,52^ The reason appears to stem from the influence of the cations on the transition state of H_2_O dissociation and its products, as mentioned by Bender et al.^45^ and Monteiro et al.^51^ Since water dissociation is rate-limiting in the case of Au, but not for Pd, a different trend emerges. Although this relationship between cation size and electrocatalytic activity has been explored for the HER in literature, a similar trend might emerge for the HOR, being the reverse reaction of the HER with the same pathways and intermediate.

**Figure 4:**
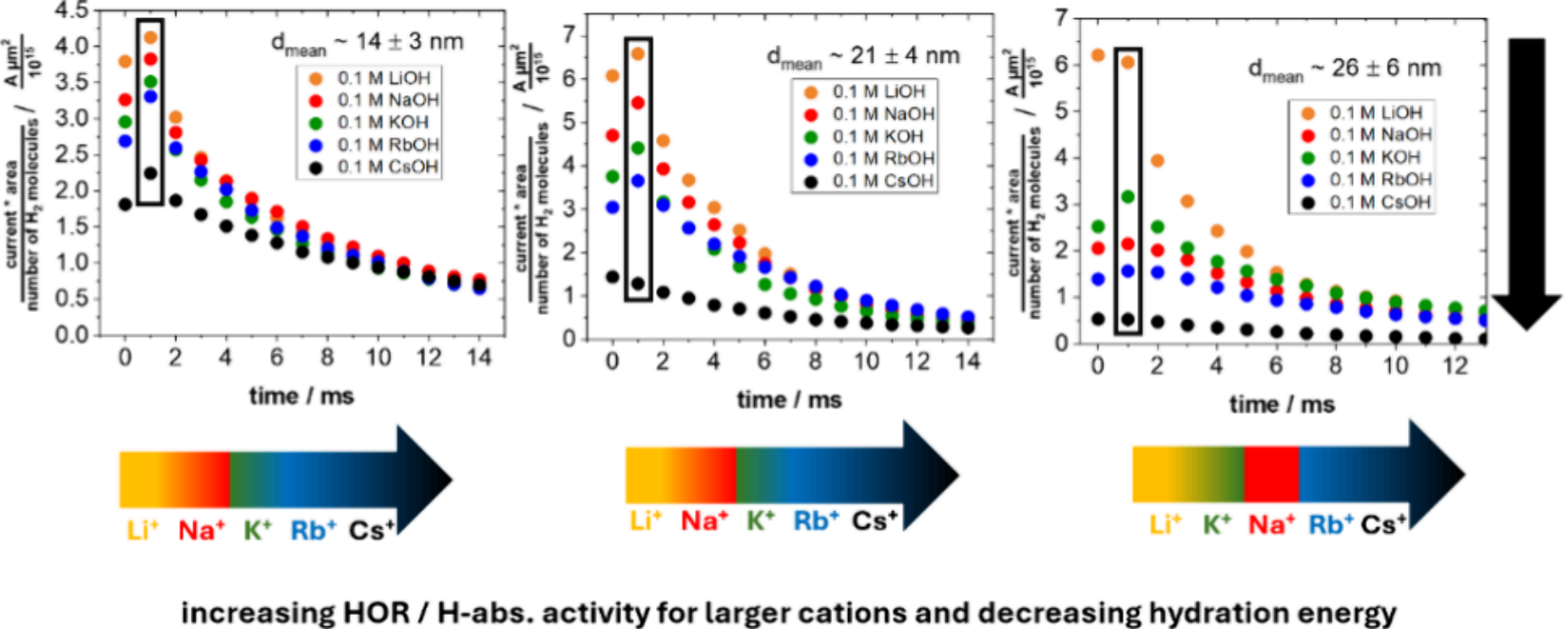
Integrated HOR microelectrode currents from HOR/H_abs_ array scans during RC-SECM experiments. Integrated and normalized HOR microelectrode currents are shown for each individual sample explored in different 0.1 M AMOH (AM = Li^+^, Na^+^, K^+^, Rb^+^, Cs^+^) electrolytes for the first 14 ms. The current was normalized by the number of hydrogen molecules produced during the third pulse.

The HOR/H_abs_ activity trend of the Pd nanopillars grown onto Au­(pc), shown in Figure 4, aligns more closely with the expected trend for Au than with Pd in 0.1 M AMOH electrolytes. From our perspective, it is most likely that the d-band center of the Pd atoms in the vicinity of the Pd/Au borders is shifted due to the different lattice parameters of Pd and the Au substrate.^13,18^ The expected trend for Au is observed for the ALD samples with 600 and 1000 cycles, which consist of numerous Pd/Au border regions due to their lower Pd coverage, as evident from the SEM image in Figure 2a. This is also supported by the XPS analysis, which revealed a (sub)­surface Pd content of 25.3% and 45.3% for the 600 and 1000 cycles, respectively (Table S1). The conducted post-mortem, cross-sectional SEM imaging in Figure S12a displays the Au surface of the sample after 600 cycles, which is clearly decorated with Pd nanopillars. The coverage of the Pd increases continuously for the 1000 (Figure S12b) and 1600 (Figure 5a) cycles. For the 1600 cycle sample, cross-sectional SEM displays a continuous but rough and porous Pd layer onto which numerous Pd nanopillars are grown. The overall thickness of this layer is approximately 45 nm with a standard deviation of ± 6 nm, due to the nanopillars making the upper part of the layer rougher. Moreover, the AFM roughness measurements of this sample (Figure 5b) compared to the uncoated Au­(pc) sample (Figure S13) confirm the presence of Pd nanopillars (with a height of approx. 10-20 nm) that are protruding from the underlying Pd layer. Due to the nanopillar diameter of ∼(26 ± 6) nm, the electrolyte still has access to the d-band shifted Pd atoms in the proximity of the Pd/Au border. The influence of these highly active Pd atoms becomes less significant since the Na^+^-containing AMOH displays a higher activity than the one with K^+^, which is in agreement with bulk Pd.

**Figure 5:**
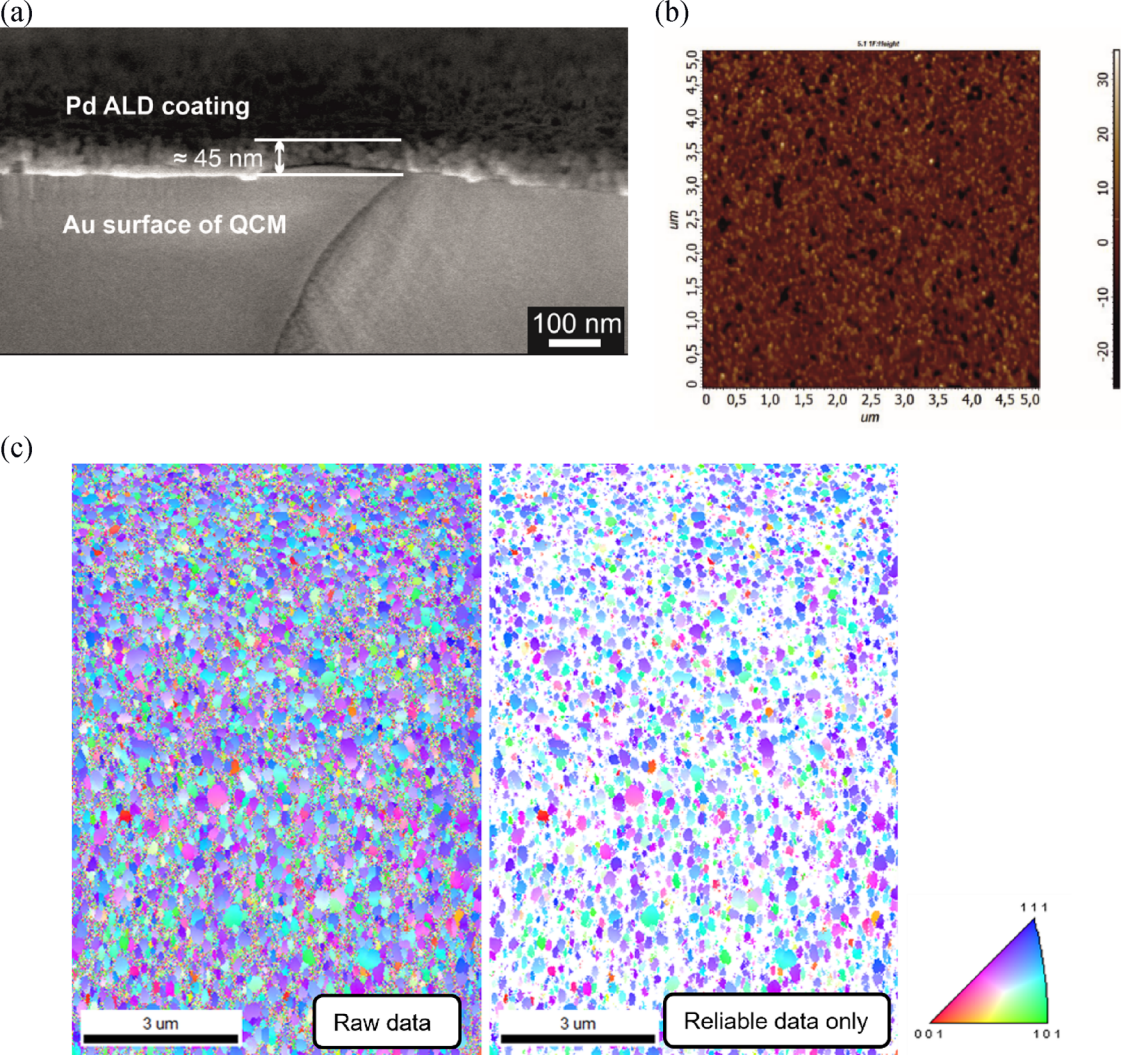
Post-mortem (a) cross-sectional SEM, (b) AFM roughness measurements, and (c) EBSD of the Pd-coated Au­(pc) sample (with 1600c) at the area of electrochemical characterization.

Furthermore, post-mortem electron backscatter diffraction mapping (EBSD) was carried out on the Pd sample with 1600 ALD cycles (Figure 5c) in comparison to the reference Au­(pc) substrate (Figure S14). The EBSD data were affected by the very fine size of the Au crystallites and, therefore, the large proportion of grain boundaries in the analyzed areas. Grain boundaries generally yield low-quality diffraction data, so only the higher confidence part of the datasets was used to generate inverse pole figure (IPF) maps (right figure). Figure 5c and Figure S14 show the amount of data disregarded because of this effect. Figure S15 illustrates the crystallographic texture of the Au and Au + Pd particles. The most prominent component in both cases is the almost perfect alignment of (111) directions with the Au­(pc) normal, i.e., the Au or Au + Pd crystallites have their (111) planes exposed in parallel with the surface. Pd deposited on the Au­(pc) seems to follow the same crystallographic orientation, validating that RC-SECM measurement did not result in significant reconstruction of the Pd nanopillars.

To further explore whether the inverted cation trend for Pd arises from the influence of the underlying Au substrate, LICT experiments were conducted to determine the so-called PME of the Pd/Au system. The PME is closely related to the orientation of interfacial water within the electrochemical double layer and corresponds to the potential at which maximal disorder occurs.^42^ Especially in alkaline media, the structure of interfacial water is believed to crucially influence reaction kinetics, as the rearrangement of water dipoles after electron transfer necessitates further energy.^42,53^ All experimental results obtained from LICT measurements suggest that the closer the PME is positioned to the thermodynamic equilibrium potential of the electrochemical reaction, the more electrocatalytically active the reaction appears to be.^54^ This indicates that the PME functions as a descriptor to predict reaction kinetics, even though a fundamental theory correlating the PME with reaction kinetics is still under development.

Since LICT experiments require smooth samples to perform experiments, and the influence of the Au substrate on Pd/Au system is most significant for thin layers, all LICT experiments were conducted with a Pd_ML_ deposited on Au­(pc) and Au(111) using the UPD method. Exemplary surface charge dependencies on the applied potential during LICT measurement in 0.1 AMOH electrolytes for the Pd_ML_/Au­(111) system are provided in Figures S16–S20. Figure 6 displays the observed PME trends depending on the employed cation in 0.1 M AMOH electrolytes for the Pd_ML_/Au­(pc) and Pd_ML_/Au­(111) samples. Both systems demonstrate a decreasing PME trend with increasing cation size and decreasing hydration energies. The closer the PME is positioned to the equilibrium potential of HER/HOR/H_abs_, the higher the activity of the Pd_ML_/Au­(pc) and Pd_ML_/Au­(111) systems in the respective electrolyte. Therefore, from LICT experiments, it can be predicted that the HER/HOR/H_abs_ activity trend corresponds to j_LiOH_ < j_NaOH_ < j_KOH_ < j_RbOH_ < j_CsOH_. These predicted activities from LICT align perfectly with the HOR/H_abs_ activity observed by SECM for the Pd nanopillars, validating our developed RC-SECM approach.

**Figure 6:**
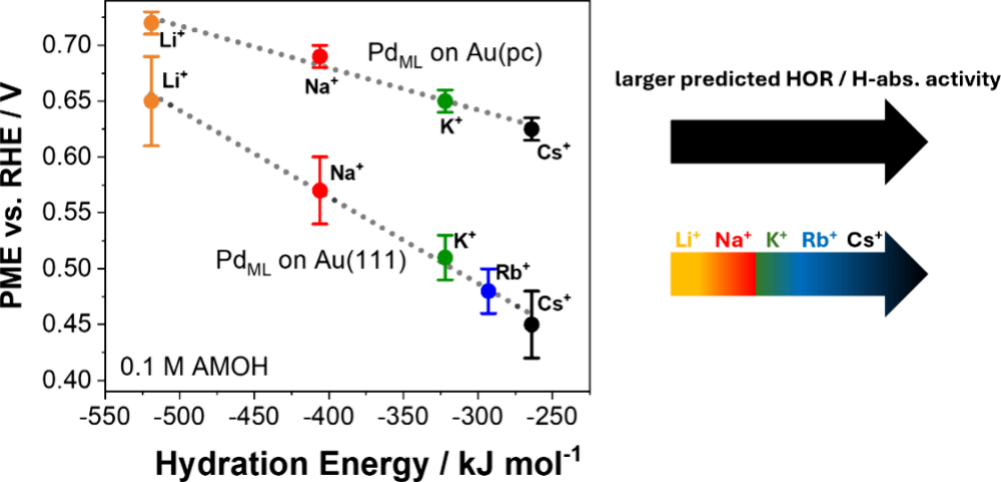
PME determined from LICT as a function of the hydration energy of alkali metal cations in 0.1 M AMOH electrolytes for Pd monolayers deposited on either Au­(pc) or Au(111). The arrows on the right indicate that the PME in 0.1 M CsOH is positioned closest to the equilibrium potential of the HER/HOR and, therefore, should exhibit the highest reaction activity.

## Conclusions

In this work, we have studied the HER activity of a sub-monolayer of Pd on Au­(pc) at the Pd/Au border using the SG-TC mode of SECM. The local kinetic assessment suggests enhanced HER activities of Pd in proximity to the Pd/Au border, which agrees with the results in the literature. Consequently, Pd/Au samples with an increasing density of such Pd/Au borders were synthesized via ALD and characterized toward the HOR using the RC-SECM mode. These measurements were conducted in 0.1 M AMOH (AM = Li^+^, Na^+^, K^+^, Rb^+^, Cs^+^) to investigate the influence of AM cations on the HOR activity. The spatial and temporal resolution of the activity via SECM reveals positions with higher activity, which expand or merge with prolonged measurement times. In addition, it is possible to quantify the localized measurement to accurately compare activities determined in different electrolytes at identical areas. From this quantification, we identify a larger HOR/H_abs_ activity for cations with smaller hydration energies: j_LiOH_ < j_NaOH_ < j_KOH_ < j_RbOH_ < j_CsOH_. The observed trend is in contrast to the dependencies reported in the literature for pure Pd and, most likely, arises due to the highly active Pd at the respective Pd/Au borders, as identified by SECM. For this reason, we performed independent LICT measurements, which display a similar dependency of the AM cations on the activity, confirming the results obtained from the RC-SECM method.

## Supplementary Material



## Notes

XPS survey and Pd 3d high-resolution spectra (600 and 1000 ALD cycle samples); exemplary current versus time profile during the third pulse of the RC-SECM procedure; number of hydrogen molecule produced during third pulse of RC-SECM for the 1600 ALD cycle Pd nanopillar sample; HOR/H_abs_ local activity maps for the 1600 ALD cycle sample and all electrolytes; video of HOR/H_abs_ local activity maps (first 5 ms, 1600 ALD cycle sample), RC-SECM mode validation, post-mortem, cross-sectional SEM, AFM, EBSD, and texture plots as pole figures for the 1600 ALD cycle sample; LICT surface charge versus applied electrode potential for Pd_ML_/Au­(111) sample in all electrolytes (PDF)
