# Supplementary material for: Revealing Catalytic Properties of Palladium/Gold Systems toward Hydrogen Evolution, Oxidation, and Absorption with Scanning Electrochemical Microscopy
Source: ACS Catal. 2025 May 14;15(11):9035–46. doi: 10.1021/acscatal.5c00783 (PMC12150267; doi:10.1021/acscatal.5c00783)
Supplement: Supplementary file 1 [file cs5c00783_si_001.pdf]

Supporting Information:

Revealing Catalytic Properties of Palladium/Gold Systems towards Hydrogen Evolution, Oxidation, and Absorption with Scanning Electrochemical Microscopy

Christian M. Schott,[1] Julia Holl,[1] Raul Zazpe,[2,3] Michael Kopp,[1] Ondřej Man,[2] Sitaramanjaneya M. Thalluri,[2,3] Jhonatan Rodriguez-Pereira,[2,3] Peter M. Schneider,[1] Kun-Ting Song,[1] Emre Keles,[1] Pekka Peljo,[4] Jerzy J. Jasielec,[4,5] Elena L. Gubanova,[,1] Jan M. Macak,*[,2,3] Aliaksandr S. Bandarenka*[,1,6]*

1 - Physics of Energy Conversion and Storage, Technical University of Munich, James Franck Str. 1, 85748 Garching, Germany

2 - Central European Institute of Technology, Brno University of Technology, Purkynova 123, 61200 Brno, Czech Republic

3 - Center of Materials and Nanotechnologies, Faculty of Chemical Technology, University of Pardubice, Nam. Cs. Legii 565, 53002 Pardubice, Czech Republic

4 -Research Group of Battery Materials and Technologies, Department of Mechanical and Materials Engineering, Faculty of Technology, University of Turku, 20014 Turun Yliopisto, Finland

5 – Department of Physical Chemistry and Modelling, Faculty of Materials Science and Ceramics, AGH University of Science and Technology, Al. Mickiewicza 30, 30-059 Kraków, Poland

6 - Catalysis Research Center TUM, Ernst-Otto-Fischer-Str. 1, 85748 Garching, Germany

* Corresponding author emails: elena.gubanova@tum.de, jan.macak@upce.cz, bandarenka@ph.tum.de

KEYWORDS scanning electrochemical microscopy; hydrogen oxidation reaction; hydrogen evolution reaction; hydride formation; monolayer; nanostructures; palladium

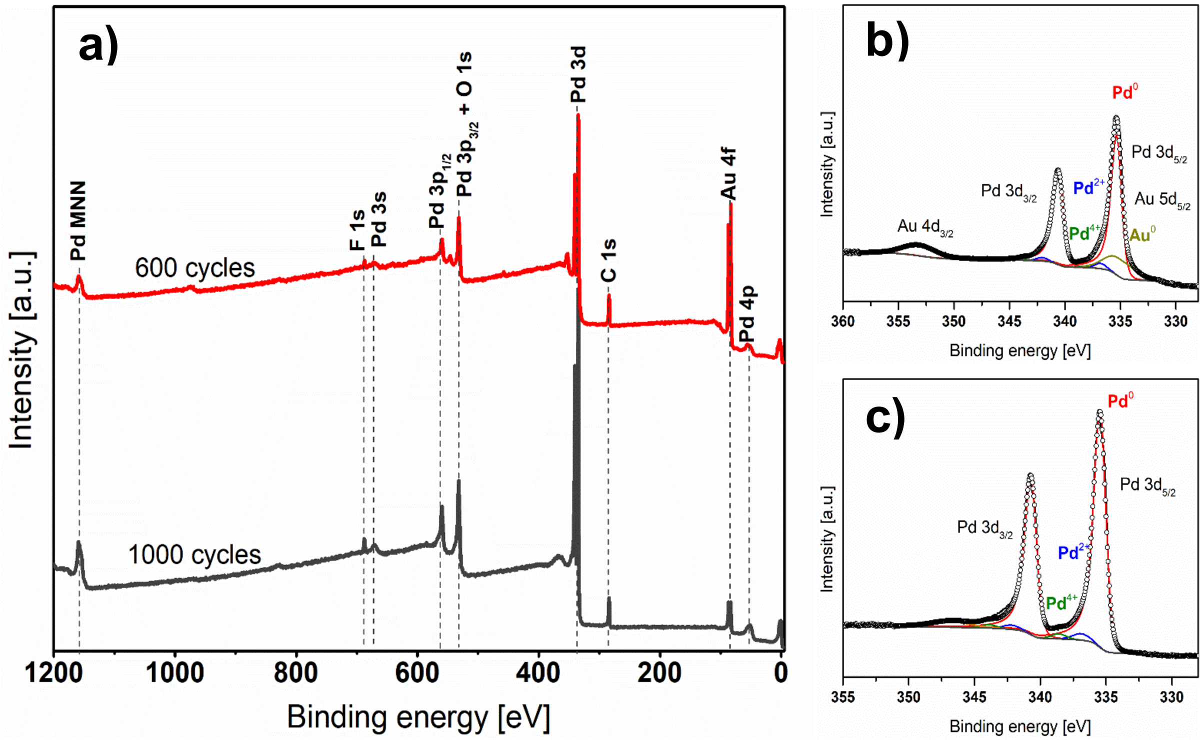

Figure S1. XPS spectra of Pd deposited on QCM by ALD: a) Survey of 600 and 1000 ALD cycles, and Pd 3d high-resolution spectra of b) 600 ALD cycles and c) 1000 ALD cycles.

Table S1. Atomic concentration (%) of elemental (sub)surface content for the Pd deposits on Au with 1000c and 600c, respectively.

**Sample**	**C**	**Pd**	**O**	**Au**	**F**
1000c	37.0	45.3	8.4	3.0	6.3
600c	41.2	25.3	16.2	13.7	3.6

Table S2. Pd atomic concentration (%) of the estimated (sub)surface composition of the Pd deposits on Au with 1000c and 600c, respectively.

**Sample**	**Pd^0**	**Pd^{2+}**	**Pd^{4+}**
1000c	95.1	2.9	2.0
600c	96.0	3.3	0.7

Redox competition (RC)-scanning electrochemical microscopy (SECM) measurements of Pd Nanopillars Grown on Au(pc)

A scheme of the RC-SECM mode was provided in **Figure 2b** in the main manuscript. A microelectrode is positioned at a distance of ~10 µm from the sample, and a series of pulses are applied, including conditioning, oxygen reduction (ORR), hydrogen evolution (HER), and hydrogen oxidation reaction (HOR). First, the conditioning pulse is applied for 1 s in a potential regime, where minor faradaic processes occur, allowing the system to recover from emerging local pH gradients in the electrolyte. During the second pulse, local oxygen is reduced in the electrolyte between the tip and the local sample position for 1 s. After the local reduction of oxygen, its diffusion toward the investigated local area is significantly hindered due to the small tip-to-sample separation distance of ~10 µm. In the third pulse, molecular H_2 is generated within the electrolyte encapsulated between the tip and the sample for 0.4 s. The time and the respective potential were empirically determined and optimized to prevent H_2 bubble formation between the microelectrode and sample. It is important to note that molecular H_2 can be produced even at slightly positive potentials versus the RHE scale due to small Nernstian potential shifts arising from the non-entirely hydrogen-saturated electrolyte. In the fourth pulse, the molecular hydrogen, induced previously during the third pulse, is consumed at the microelectrode via HOR. The activity of HOR and H absorption at the sample is studied by applying an HOR/H absorption potential to the sample of interest during all mentioned microelectrode pulses. During the fourth pulse of 400 ms, the sample and the microelectrode compete to consume the previously induced molecular hydrogen. A small HOR microelectrode current indicates that the local area of the catalyst sample successfully competes against the microelectrode and consumes a significant amount of the induced molecular hydrogen. Conversely, a large HOR microelectrode current suggests a less active area of the sample, as the majority of the induced molecular hydrogen is consumed at the microelectrode.

We can use this approach to differentiate areas of the Pd/Au sample with distinct electrocatalytic activity by monitoring the HOR current recorded at the microelectrode. By careful design of the experiment and reasonable data treatment, it is possible to create a HOR/H absorption activity map of the sample for each millisecond of the HOR microelectrode pulse. However, the HOR current during the fourth pulse declines rapidly after several milliseconds since the amount of previously

induced molecular H_2 is small. These activity maps allow for the time-dependent visualization of differences in the local electrocatalytic activity. The resolution of the local electrocatalytic activity depends on several factors, such as the diameter of the employed microelectrode and tip-to-sample distance.

In the following, we would like to discuss the data treatment during the RC-SECM mode to ensure that measurements conducted in different electrolytes are comparable with each other. The RC-SECM mode was initially validated by investigating the HOR/H absorption activities in 0.1 M $HClO_4$ for the three Pd nanopillar samples with distinct diameters (14 ± 3 nm, 21 ± 4 nm, and 26 ± 6 nm) and Pd content. This validation will be discussed afterwards. Then, HOR/H absorption activities were examined in 0.1 M AMOH (AM = Li^+, Na^+, K^+, Rb^+, Cs^+) electrolytes for each Pd nanopillar sample individually to observe the influence of the cations on the activity. Here, we present the results obtained for the sample with 26 ± 6 nm-sized Pd nanopillar. Similar results were obtained for the other two samples.

It is essential to consider the amount of molecular H_2 produced during the third microelectrode pulse, which directly influences the HOR current during the fourth pulse at the microelectrode and the HOR/H absorption current at the local sample position. Therefore, we integrate the HER microelectrode current during the third pulse over time to obtain the charge Q_{HER}. This charge represents all the electrons transferred to produce molecular hydrogen within the 400 ms duration of the third pulse. For completeness, we assume that the Faradaic current during the third pulse arises purely from the HER, given that all oxygen was removed during the second microelectrode pulse. By dividing Q_{HER} by twice the electron charge (which is required to produce one hydrogen molecule), we determine the number of hydrogen molecules at the specific position of the microelectrode. **Figure S2** displays the current versus time profile during the third pulse at an arbitrary position during the RC-SECM experiments.

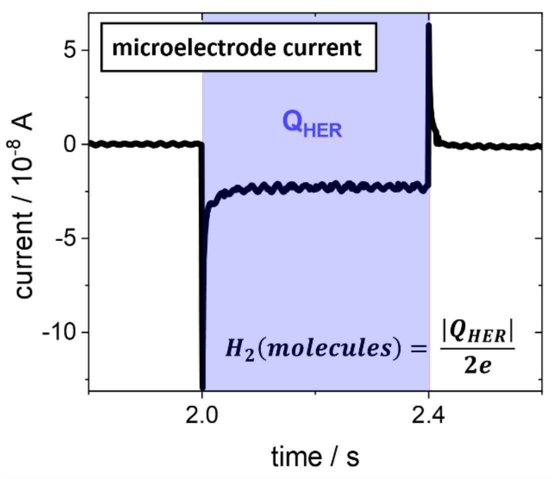

Figure S2. Microelectrode current profile during the RC-SECM experiments. The highlighted time interval from 2 to 2.4 seconds corresponds to the third microelectrode pulse during which the hydrogen evolution reaction is taking place. The current can be integrated over time to obtain the charge Q_{HER}, from which the number of hydrogen molecules can be calculated.

By evaluating this current profile at each increment of the array scan, heat maps can be created, depicting the number of hydrogen molecules at each lateral position for each individual electrolyte. These heat maps are displayed in **Figure S3a – S3f**. Significant differences occur between different electrolytes but also within a single electrolyte. These differences suggest that the quantity of produced H_2 from the Pt microelectrode depends on three factors. First, the electrolyte itself influences the number of produced hydrogen molecules, as it is well known that the HER for Pt is drastically more active in acidic (0.1 M $HClO_4$) media compared to alkaline media (0.1 M AMOH).[1] Additionally, the utilized cation in AMOH electrolytes is known to influence HER activity due to the different hydration energies of the cation, affecting interfacial water and reaction kinetics.[2] Second, during all microelectrode pulses, the HOR/H absorption potential is applied to the Pd/Au sample. Due to the continuous consumption of molecular H_2 at the sample, the number of H_2 molecules produced at the tip can vary according to the electrocatalytic HOR/H absorption activity of the local sample position. Third, the microelectrode-to-sample distance, and therefore the encapsulated electrolyte volume between tip and sample, significantly influences the amount of produced hydrogen molecules, as indicated by the variations within an individual heat map (**Figure S3**). The difference in tip-to-sample distance likely arises from sample tilt, inhomogeneities in Pd nanopillar distribution, or morphological surface changes. To correct the influence of produced H_2 molecules on the measured HOR current during the fourth pulse, the

microelectrode HOR current recorded at each individual measurement increment needs to be normalized by the previously induced number of H_2 molecules produced at the same increment. Subsequently, the normalized HOR microelectrode current at each position can be plotted over time into an activity map. It is only reasonable to create HOR activity maps for the first couple of milliseconds since the molecular H_2 concentration decreases with HOR measurement time.

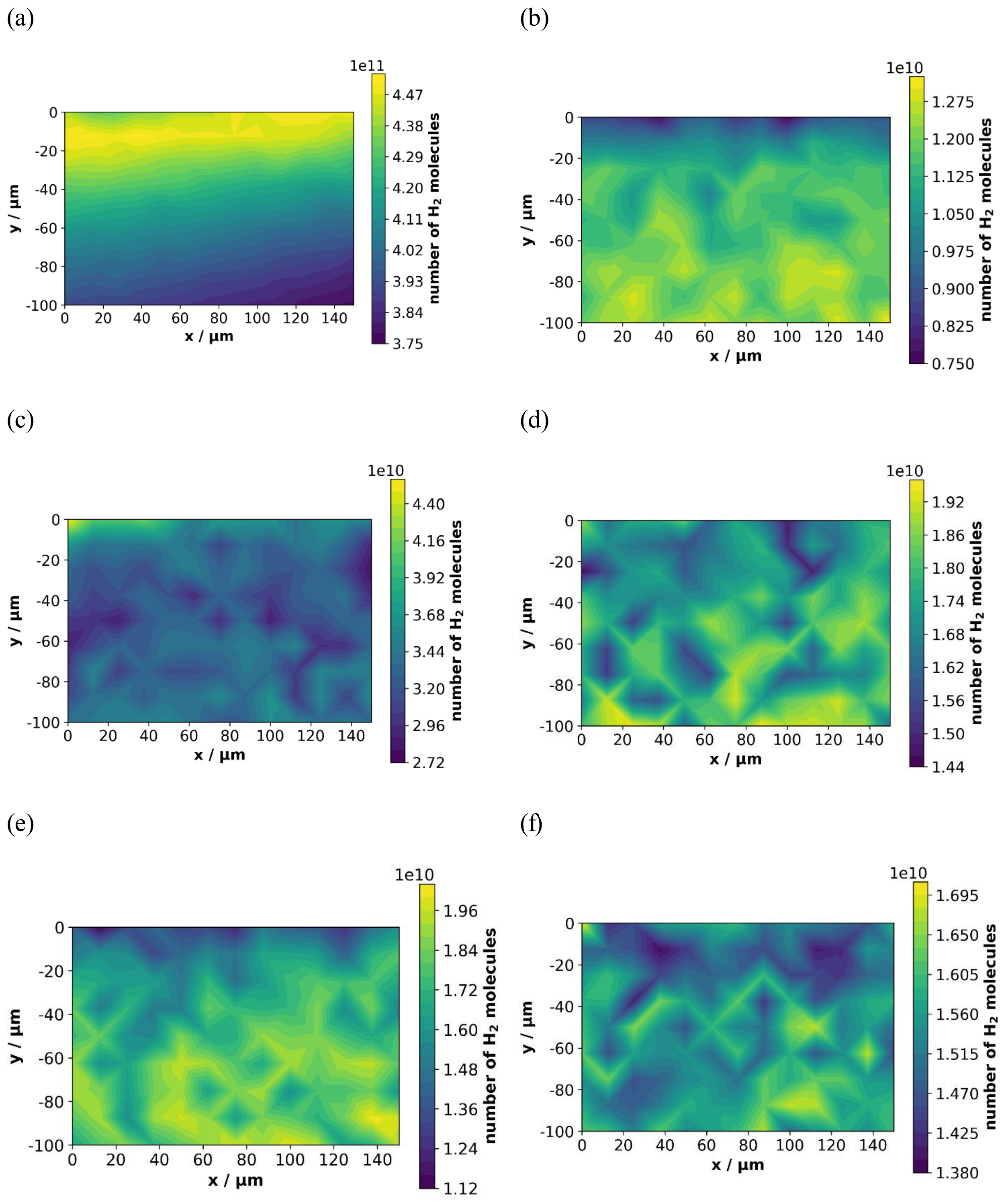

Figure S3. Number of hydrogen molecules produced during the third pulse for the sample with Pd nanopillars of 26 ± 6 nm size in (a) 0.1 M $HClO_4$, (b) 0.1 M LiOH, (c) 0.1 M NaOH, (d) 0.1 M KOH, (e) 0.1 M RbOH, and (f) 0.1 M CsOH at the same position.

Local activity maps in 0.1 M AMOH electrolytes

Local activity maps are shown for all measurements conducted on the sample with 26 ± 6 nm-sized Pd nanopillars. Each figure shows the normalized HOR microelectrode current for the first 5 ms in 0.1 M $HClO_4$ (**Figure S4**) and 0.1 M AMOH electrolytes (**Figures S5–S9**). The colors of the heat maps are consistently chosen using identical scale bars. The videos in **Figure S10** for the respective electrolytes provide a summary of all measured activity maps.

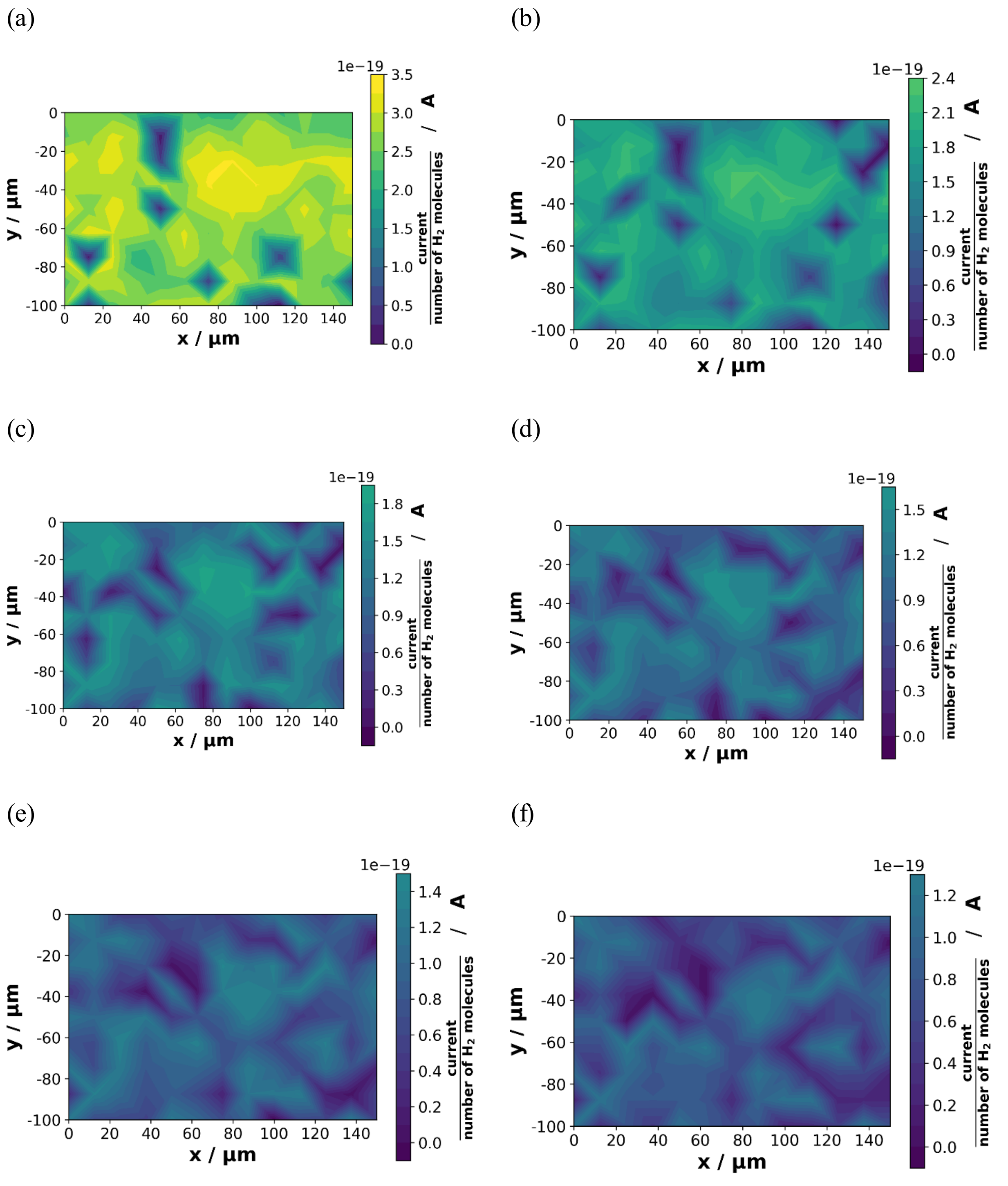

Figure S4. HOR/H absorption local activity maps obtained through RC-SECM. The heat maps display the HOR microelectrode current normalized by the previously induced hydrogen molecules. The activity maps correspond to the sample with Pd nanopillars of 26 ± 6 nm size in 0.1 M $HClO_4$ after (a) 0 ms, (b) 1 ms, (c) 2 ms, (d) 3 ms, (e) 4 ms, and (f) 5 ms of the fourth pulse.

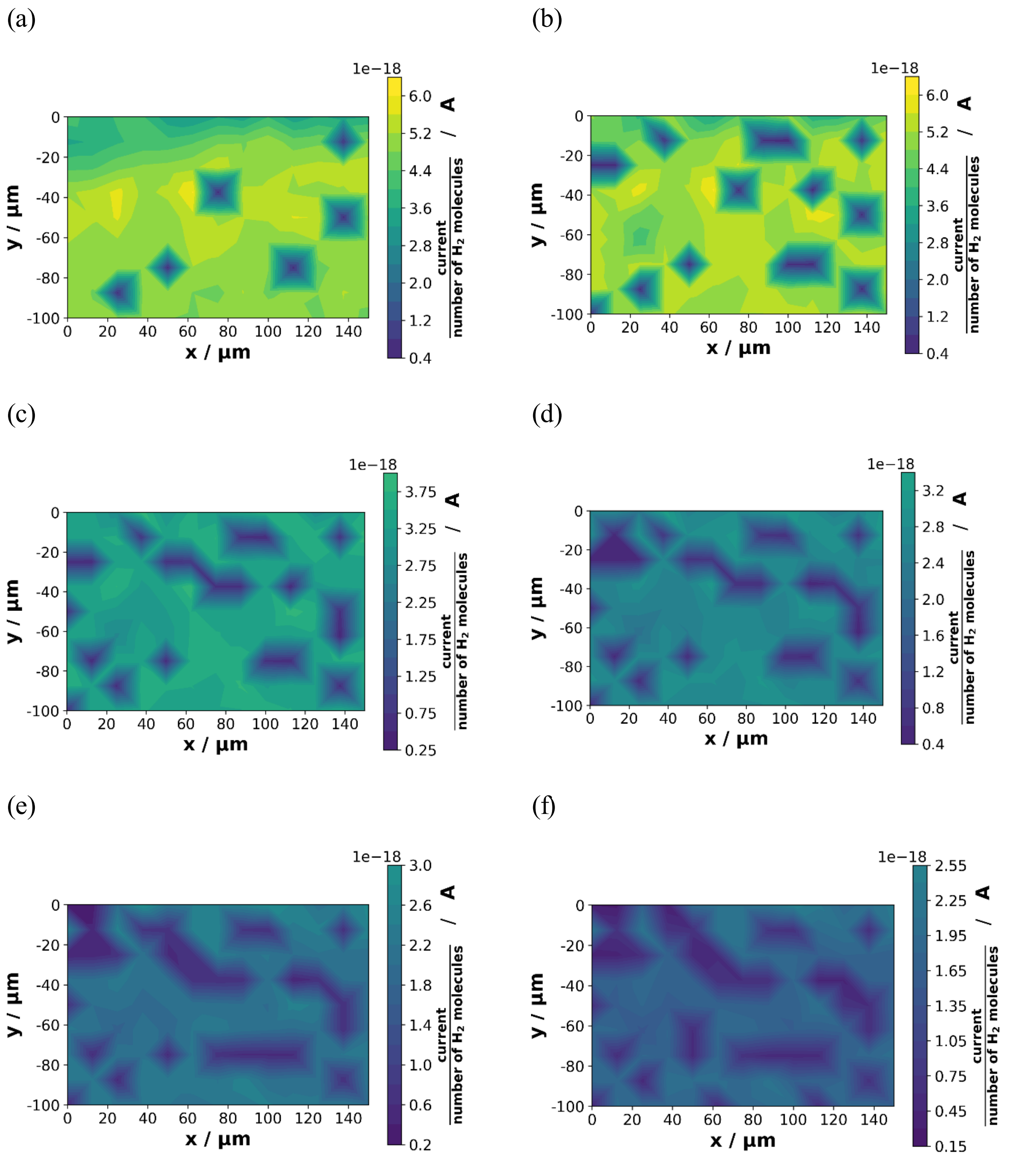

Figure S5. HOR/H absorption local activity maps obtained through RC-SECM. The heat maps display the HOR microelectrode current normalized by the previously induced hydrogen molecules. The activity maps correspond to the sample with Pd nanopillars of 26 ± 6 nm size in 0.1 M LiOH after (a) 0 ms, (b) 1 ms, (c) 2 ms, (d) 3 ms, (e) 4 ms, and (f) 5 ms of the fourth pulse.

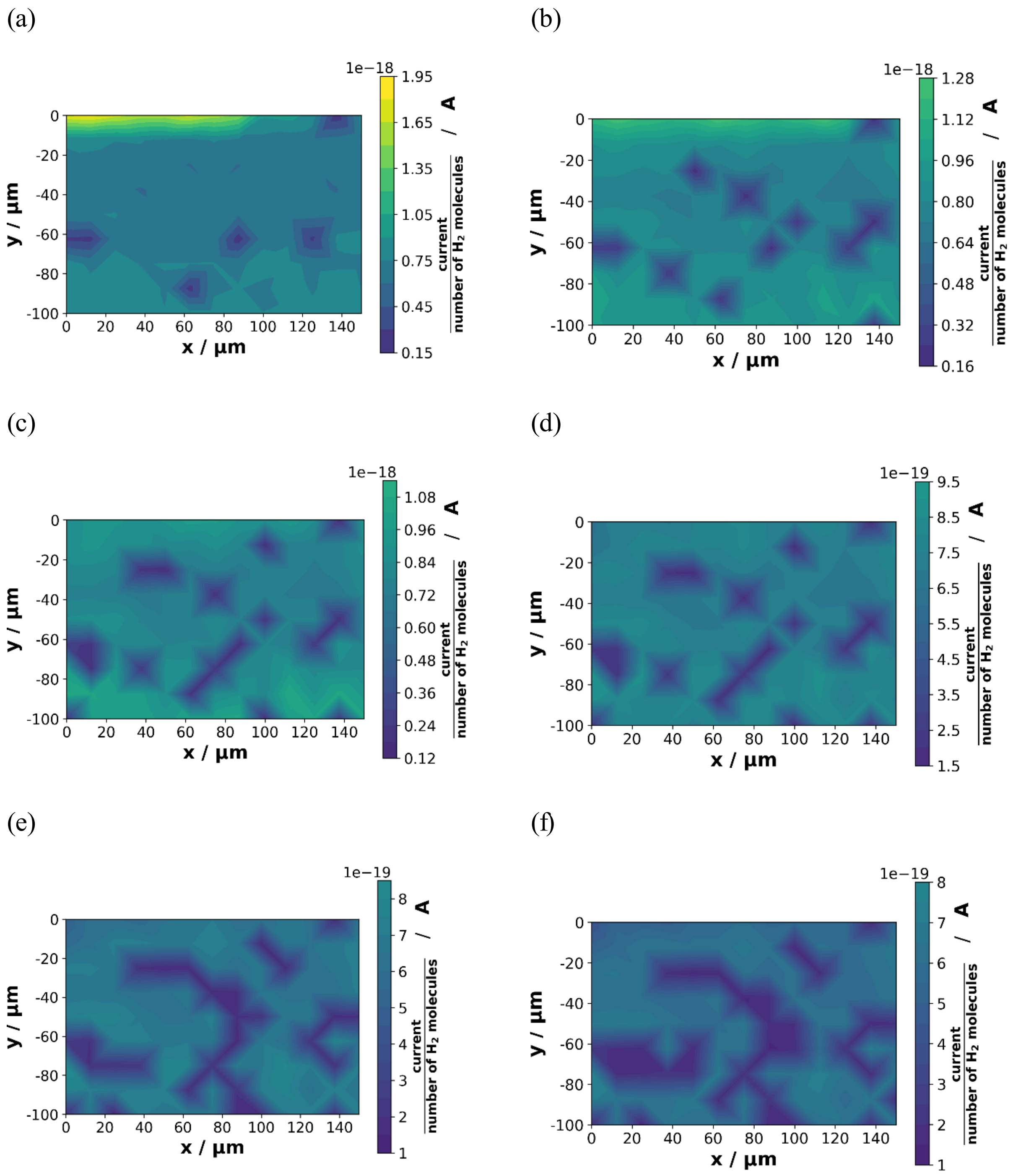

Figure S6. HOR/H absorption local activity maps obtained through RC-SECM. The heat maps display the HOR microelectrode current normalized by the previously induced hydrogen molecules. The activity maps correspond to the sample with Pd nanopillars of 26 ± 6 nm size in 0.1 M NaOH after (a) 0 ms, (b) 1 ms, (c) 2 ms, (d) 3 ms, (e) 4 ms, and (f) 5 ms of the fourth pulse.

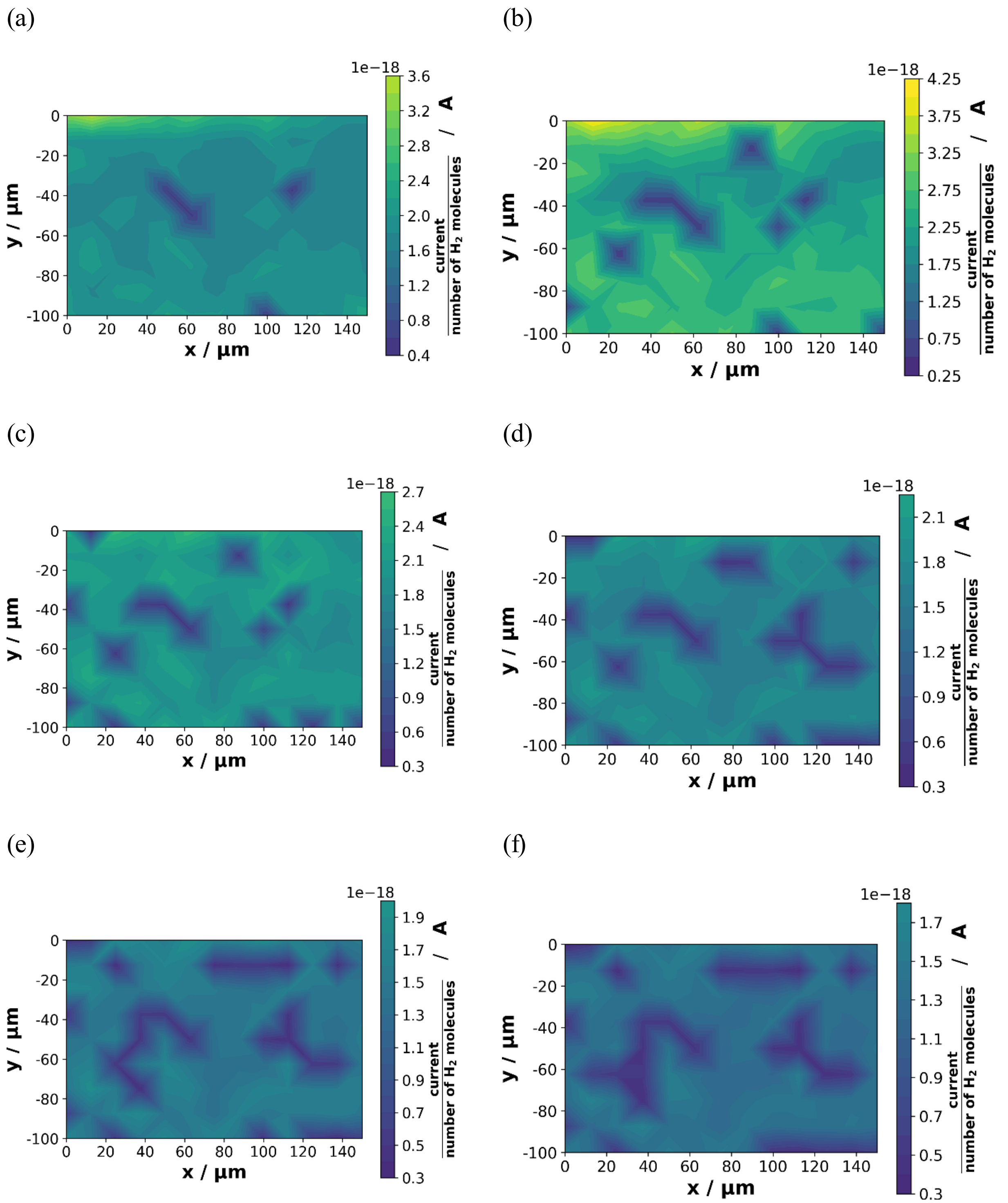

Figure S7. HOR/H absorption local activity maps obtained through RC-SECM. The heat maps display the HOR microelectrode current normalized by the previously induced hydrogen molecules. The activity maps correspond to the sample with Pd nanopillars of 26 ± 6 nm size in 0.1 M KOH after (a) 0 ms, (b) 1 ms, (c) 2 ms, (d) 3 ms, (e) 4 ms, and (f) 5 ms of the fourth pulse.

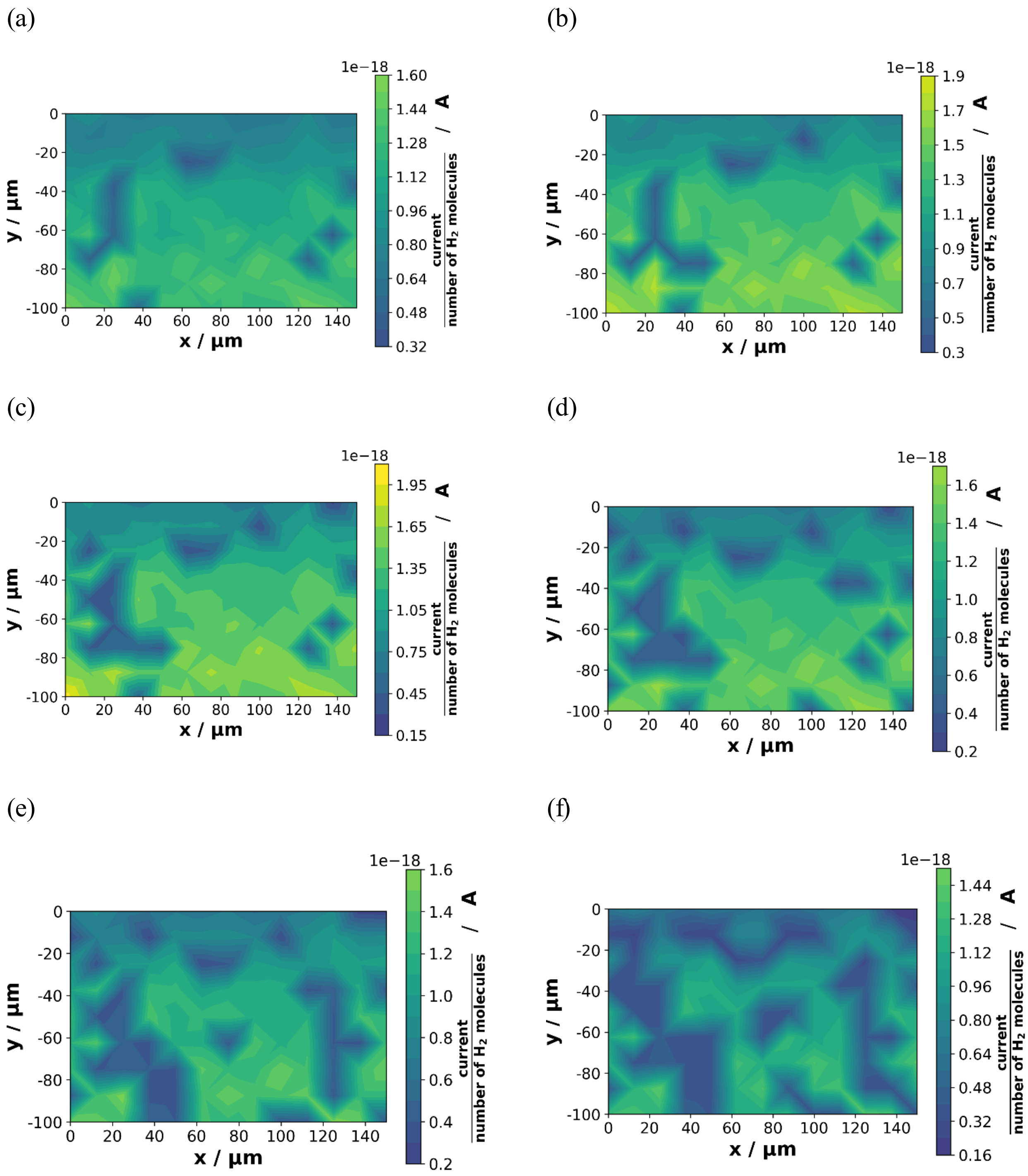

Figure S8. HOR/H absorption local activity maps obtained through RC-SECM. The heat maps display the HOR microelectrode current normalized by the previously induced hydrogen molecules. The activity maps correspond to the sample with Pd nanopillars of 26 ± 6 nm size in 0.1 M RbOH after (a) 0 ms, (b) 1 ms, (c) 2 ms, (d) 3 ms, (e) 4 ms, and (f) 5 ms of the fourth pulse.

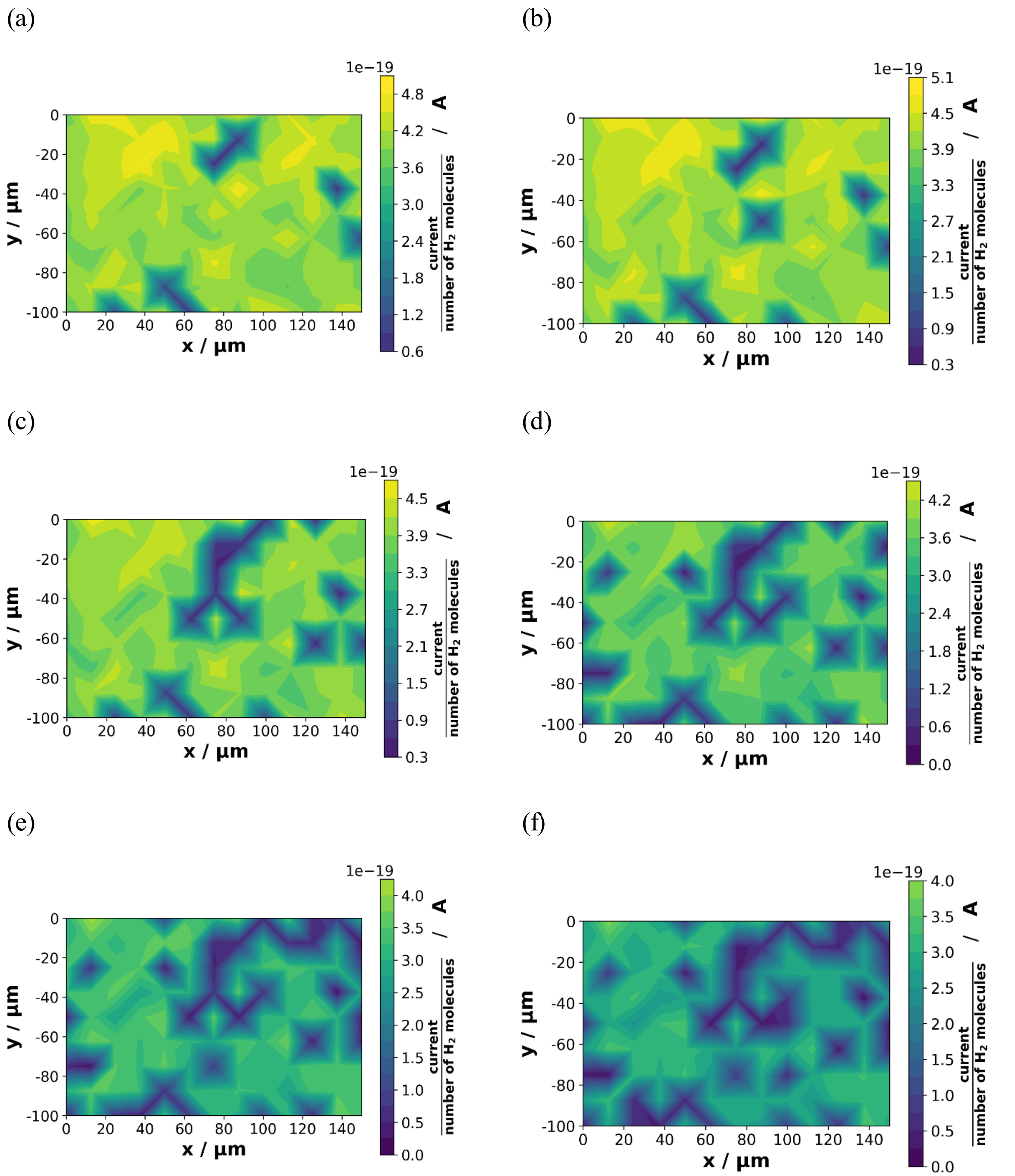

Figure S9 HOR/H absorption local activity maps obtained through RC-SECM. The heat maps display the HOR microelectrode current normalized by the previously induced hydrogen molecules. The activity maps correspond to the sample with Pd nanopillars of 26 ± 6 nm size in 0.1 M CsOH after (a) 0 ms, (b) 1 ms, (c) 2 ms, (d) 3 ms, (e) 4 ms, and (f) 5 ms of the fourth pulse.

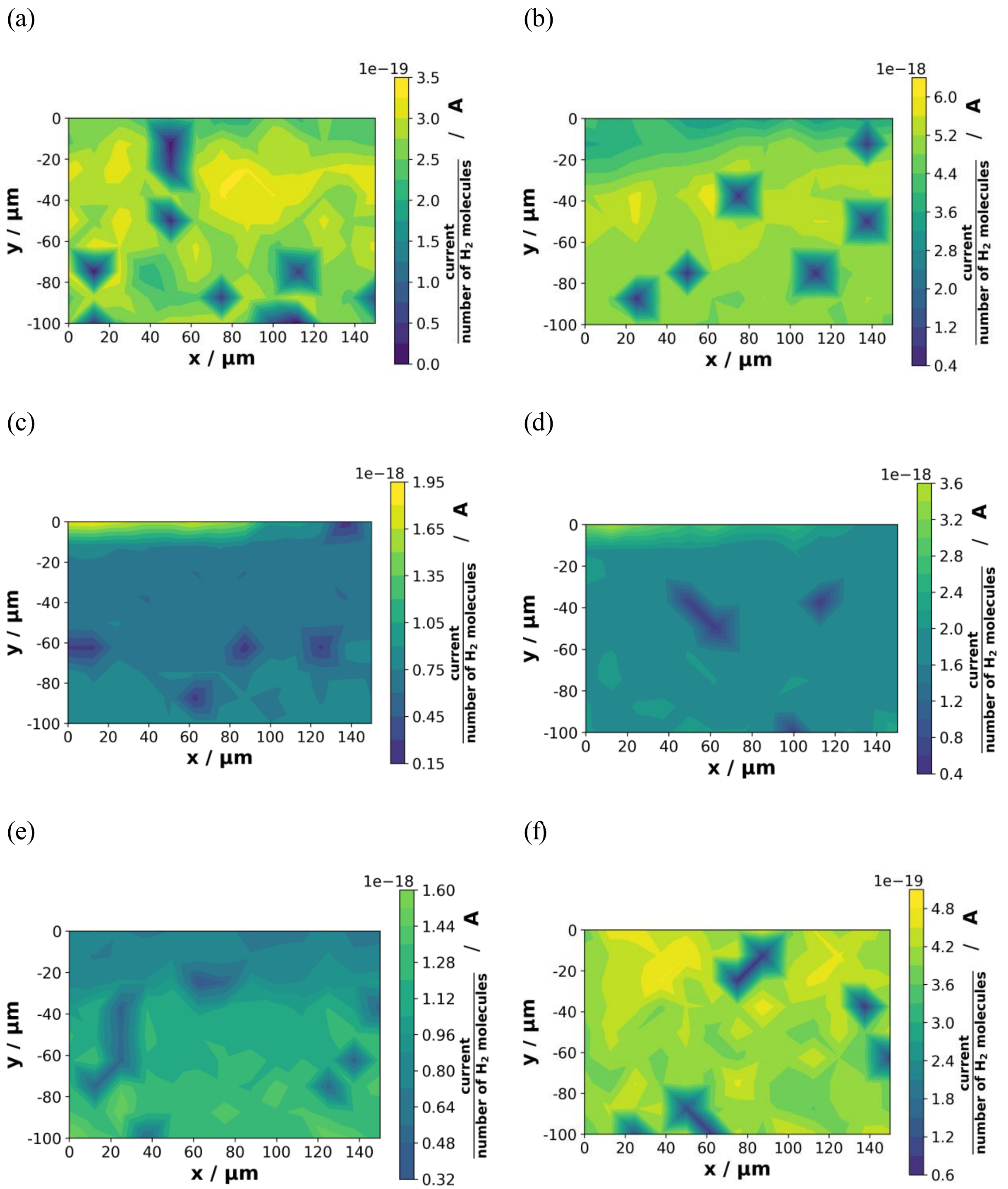

Figure S10. Videos of HOR/H absorption local activity maps obtained for the first 5 ms for the sample with Pd nanopillars of 26 ± 6 nm size in (a) 0.1 M $HClO_4$, (b) 0.1 M LiOH, (c) 0.1 M NaOH, (d) 0.1 M KOH, (e) 0.1 M RbOH, and (f) 0.1 M CsOH. The videos are created from the heat maps shown in **Figures S3–S8**.

RC-SECM mode validation

To validate this characterization concept, HOR/H absorption activity evaluations of the three different Pd nanopillar samples were conducted in 0.1 M $HClO_4$ (**Figure S11**). Due to the nanopillar growth via ALD, controlling their size and Pd content independently appears to be impossible. This means that samples comprised of larger nanopillars inherently contain higher Pd content, as suggested by the XPS analysis in **Tables S1** and **S2**, such as the post-mortem, cross-sectional SEM images in **Figure 5a**, and **Figure S12**.

However, comparing the three Pd nanopillar samples with distinct sizes and Pd content is ideal for proving the developed HOR/H absorption activity evaluation procedure, as samples consisting of larger nanopillars with higher Pd content are expected to exhibit greater HOR/H absorption activity. **Figure S11** shows the values obtained for the surface integration of the normalized HOR microelectrode current for the first milliseconds of the three Pd nanopillar samples. A clear activity trend emerges, revealing that higher HOR/H absorption activities are achieved for samples with larger Pd nanopillars and higher Pd content, as predicted above, validating the developed HOR/H absorption activity procedure using SECM.

Since molecular hydrogen present in the electrolyte between the microelectrode and the sample decreases rapidly, only parameter values obtained from the surface integration after the first millisecond were investigated.

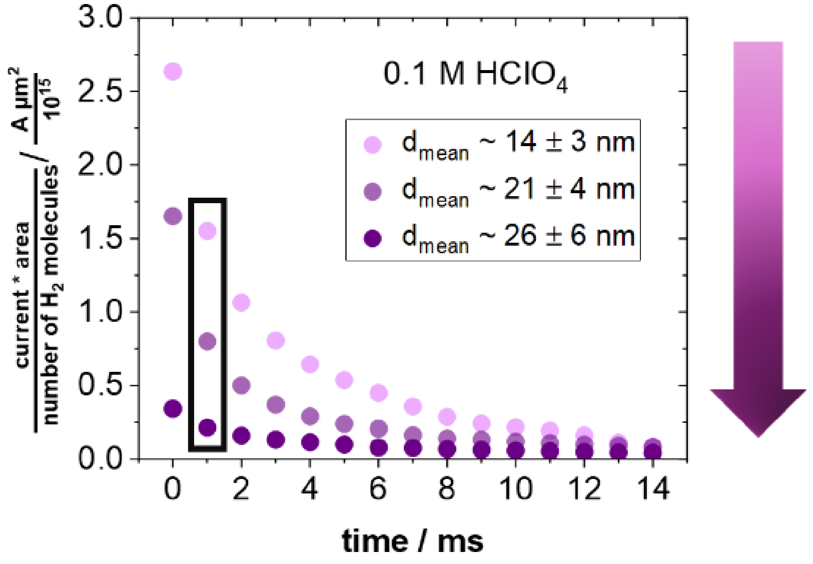

Figure S11. *Integrated HOR microelectrode currents from HOR/H abs array scans during RC-SECM experiments. The current was normalized by the number of hydrogen molecules produced during the third pulse. Integrated and normalized HOR microelectrode current for Pd nanopillar samples with different sizes and Pd content in 0.1 M $HClO_4$ for the first 14 m.*

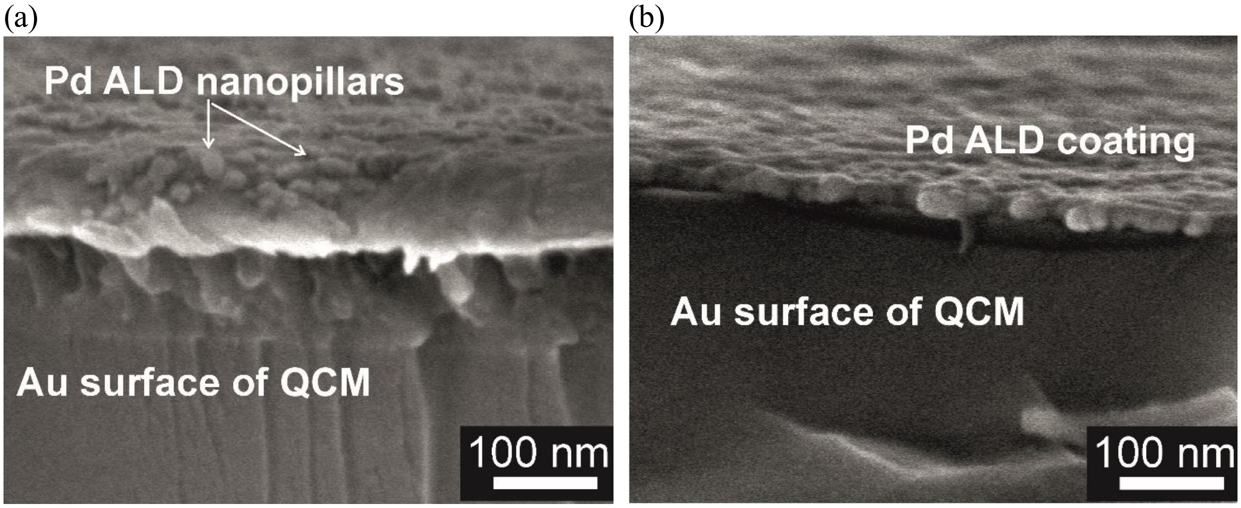

Figure S12. Post-mortem, cross-sectional SEM image of the Pd-coated Au(pc) with (a) 600 and (b) 1000 ALD cycles.

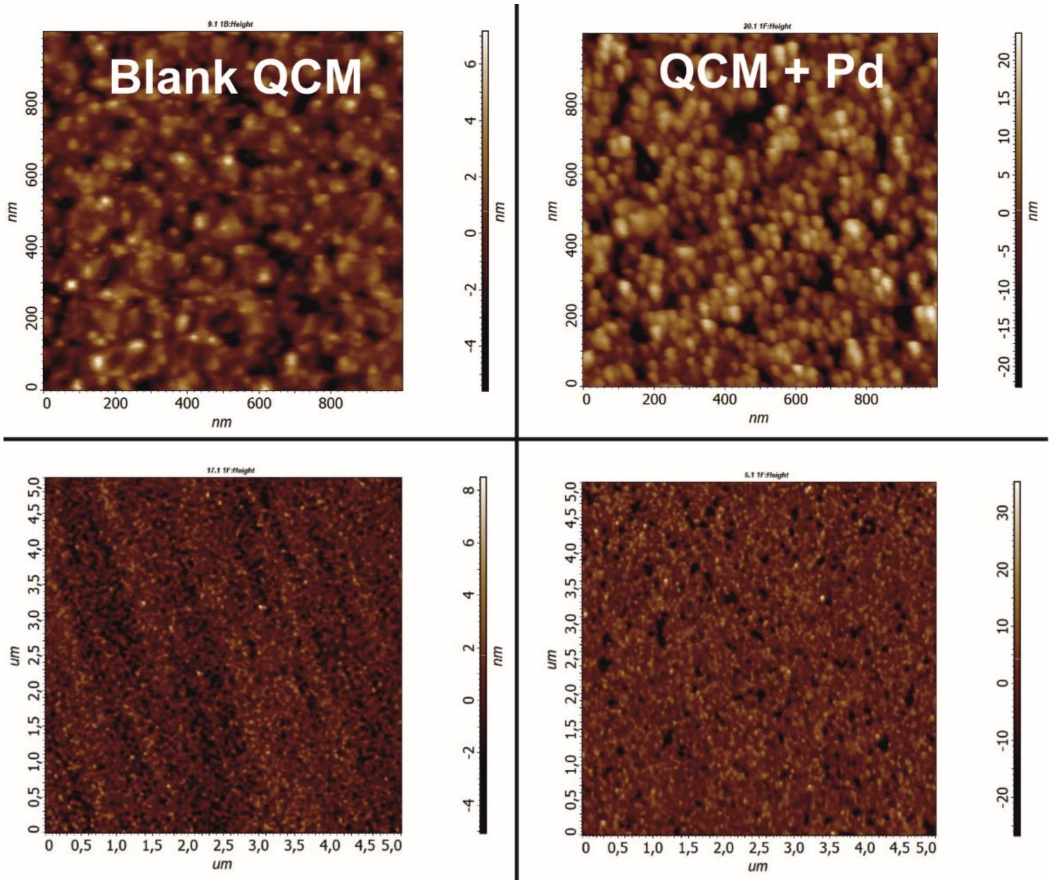

Figure S13. AFM scans (different scanned magnifications) over blank QCM crystal and Pd-QCM crystal (1600 ALD cycles).

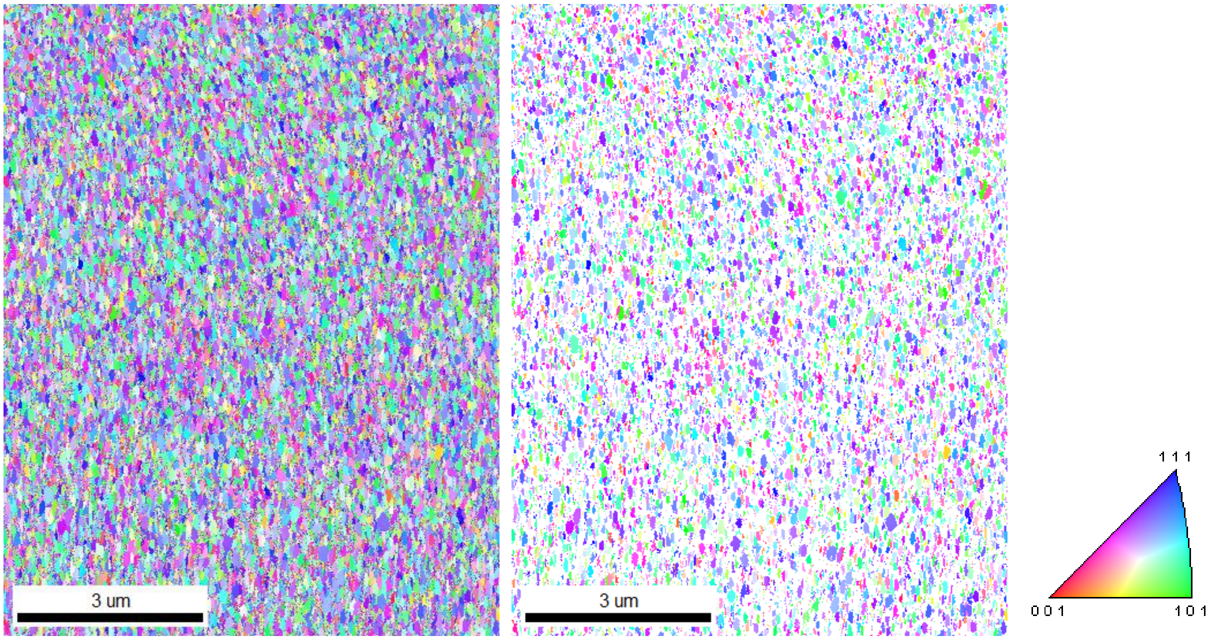

Figure S14. Inverse pole figure (IPF) maps constructed for normal direction (ND) of the Au QCM crystal. The leftmost images contain all treated data, the central ones contain only data with sufficient confidence level. Color-coding basic stereographic triangle is on the very right.

(a)

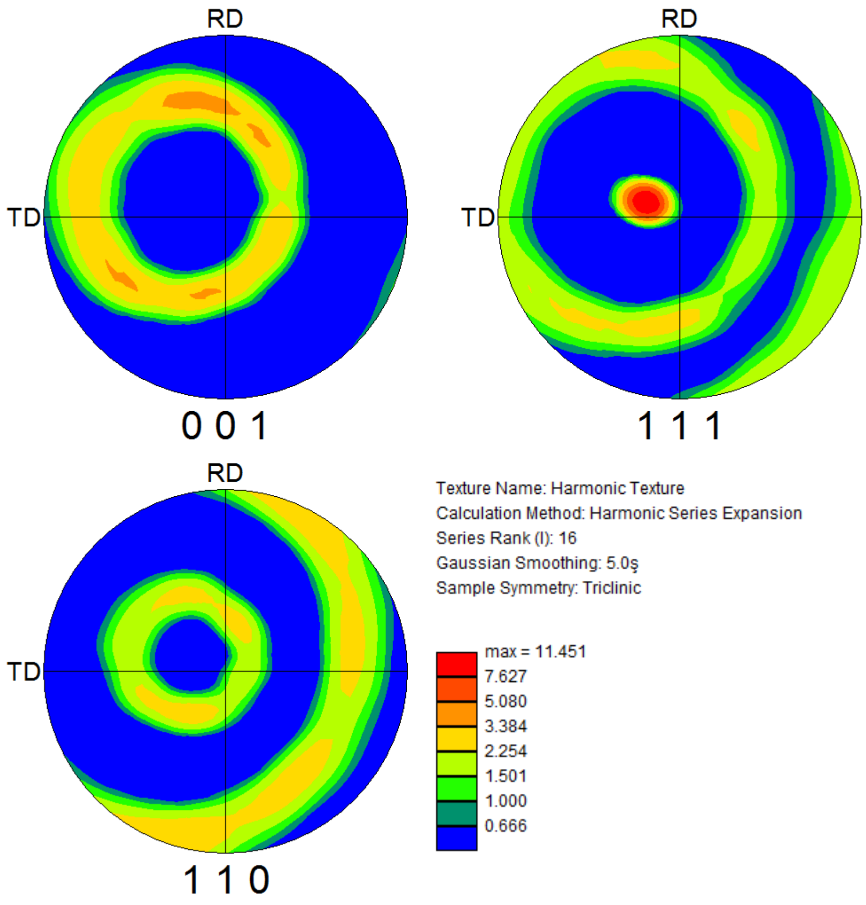
RD
TD
0 0 1
RD
TD
1 1 1
RD
TD
1 1 0
Texture Name: Harmonic Texture
Calculation Method: Harmonic Series Expansion
Series Rank (l): 16
Gaussian Smoothing: 5.0ş
Sample Symmetry: Triclinic
max = 11.451
7.627
5.080
3.384
2.254
1.501
1.000
0.666

(b)

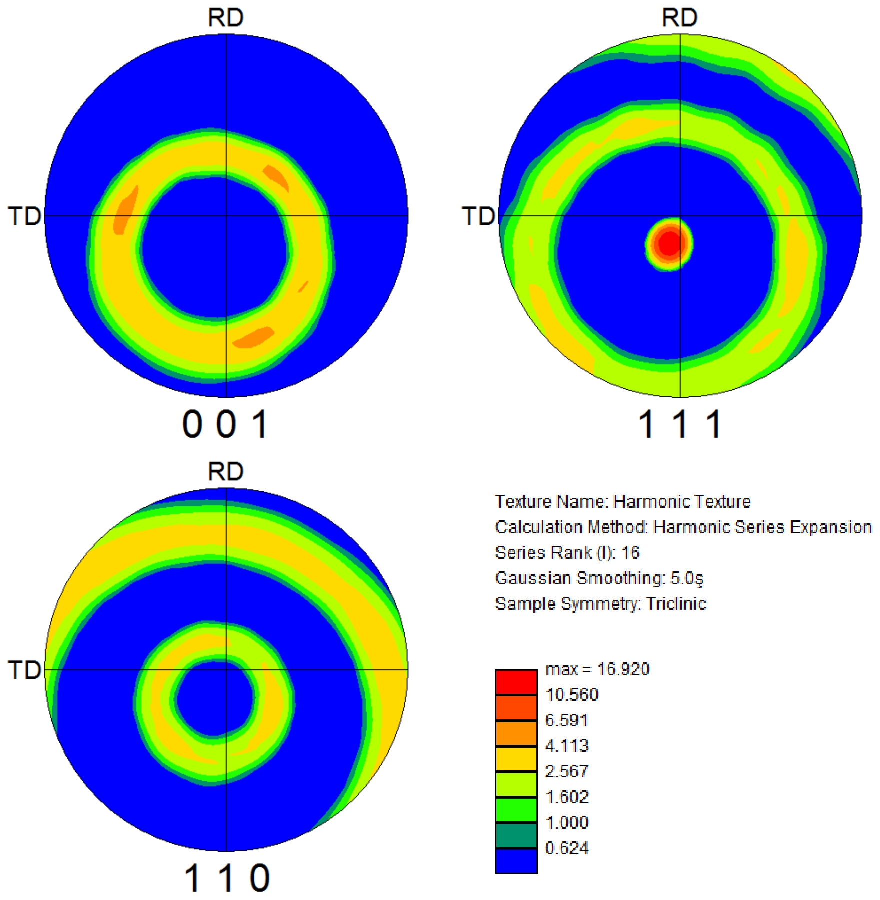

Figure S15. Texture plots as pole figures constructed for [001], [110] and [111] crystallographic directions of the (a) Au QCM crystal and (b) Pd coated one. The plots indicate almost perfect alignment of [111] planes in both crystals with normal direction (ND) to the crystal plane. The Au/Pd crystallites are otherwise rotated mostly randomly about the ND direction.

Laser-induced current transient (LICT) measurements of Pd_{ML} on Au(111)

LICT measurements were performed on a Pd_{ML} deposited onto Au(111) and Au(pc) to determine the potential of maximum entropy (PME) in 0.1 M AMOH (AM = Li^+, Na^+, K^+, Rb^+, Cs^+) electrolytes. Here, we present the determined surface charges as a function of the applied electrode potential for the Pd_{ML}/Au(111) system. Similar results were obtained for the Pd monolayer deposited on Au(pc). **Figures S16–S20** display the surface charges as a function of the electrode potential for the respective 0.1 M AMOH (AM = Li^+, Na^+, K^+, Rb^+, Cs^+) electrolytes.

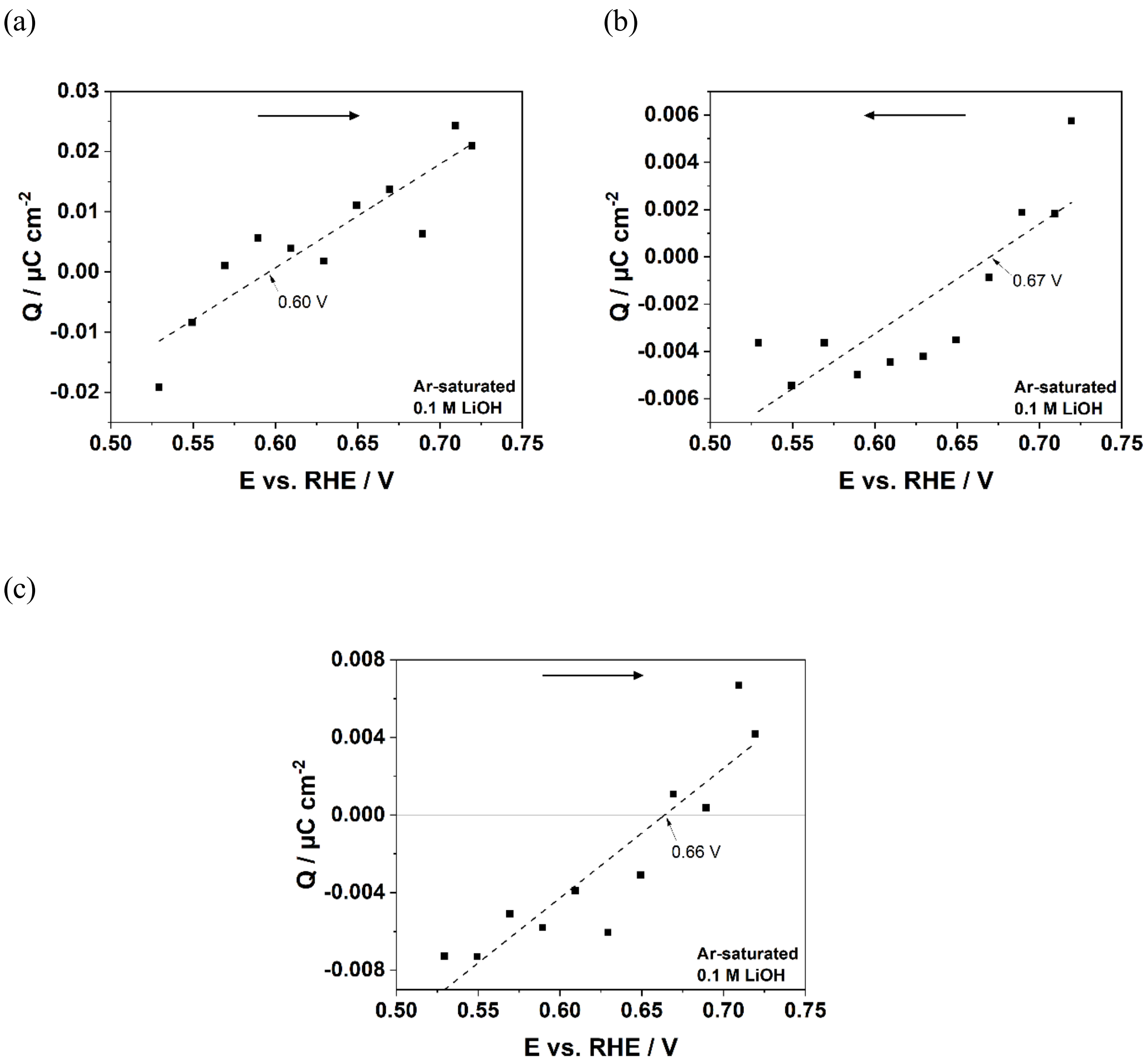

Figure S16. Surface charge as a function of the applied electrode potential for the Pd_{ML}/Au(111) working electrode in 0.1 M LiOH. The transition point between negative and positive surface charge corresponds to the PME. The respective black arrows in (a-c) display the direction of the potential sweep in the experiment.

(a)

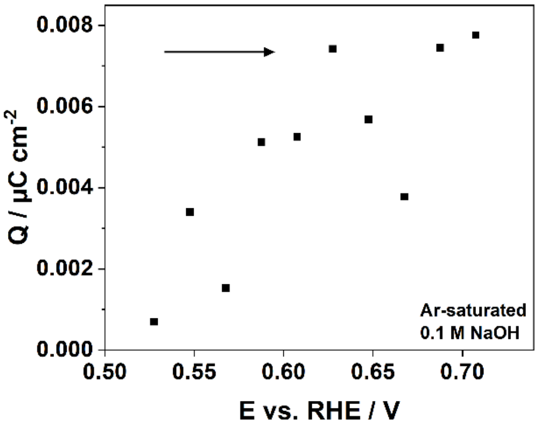

(b)

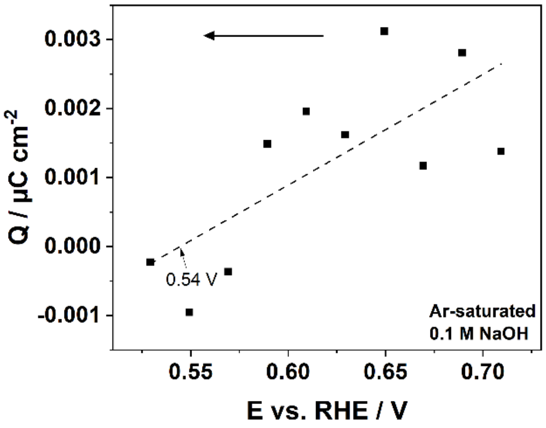

(c)

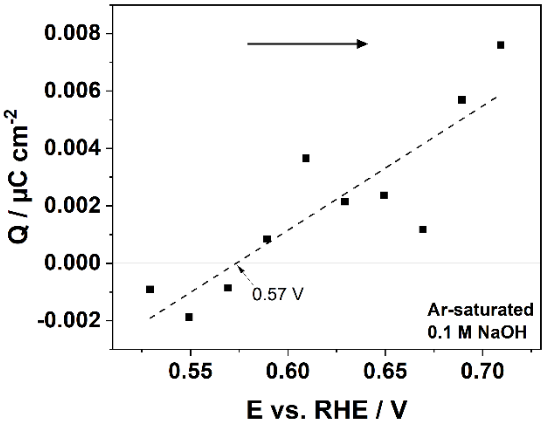

Figure S17. Surface charge as a function of the applied electrode potential for the Pd_{ML}/Au(111) working electrode in 0.1 M NaOH. The transition point between negative and positive surface charge corresponds to the PME. The respective black arrows in (a-c) display the direction of the potential sweep in the experiment.

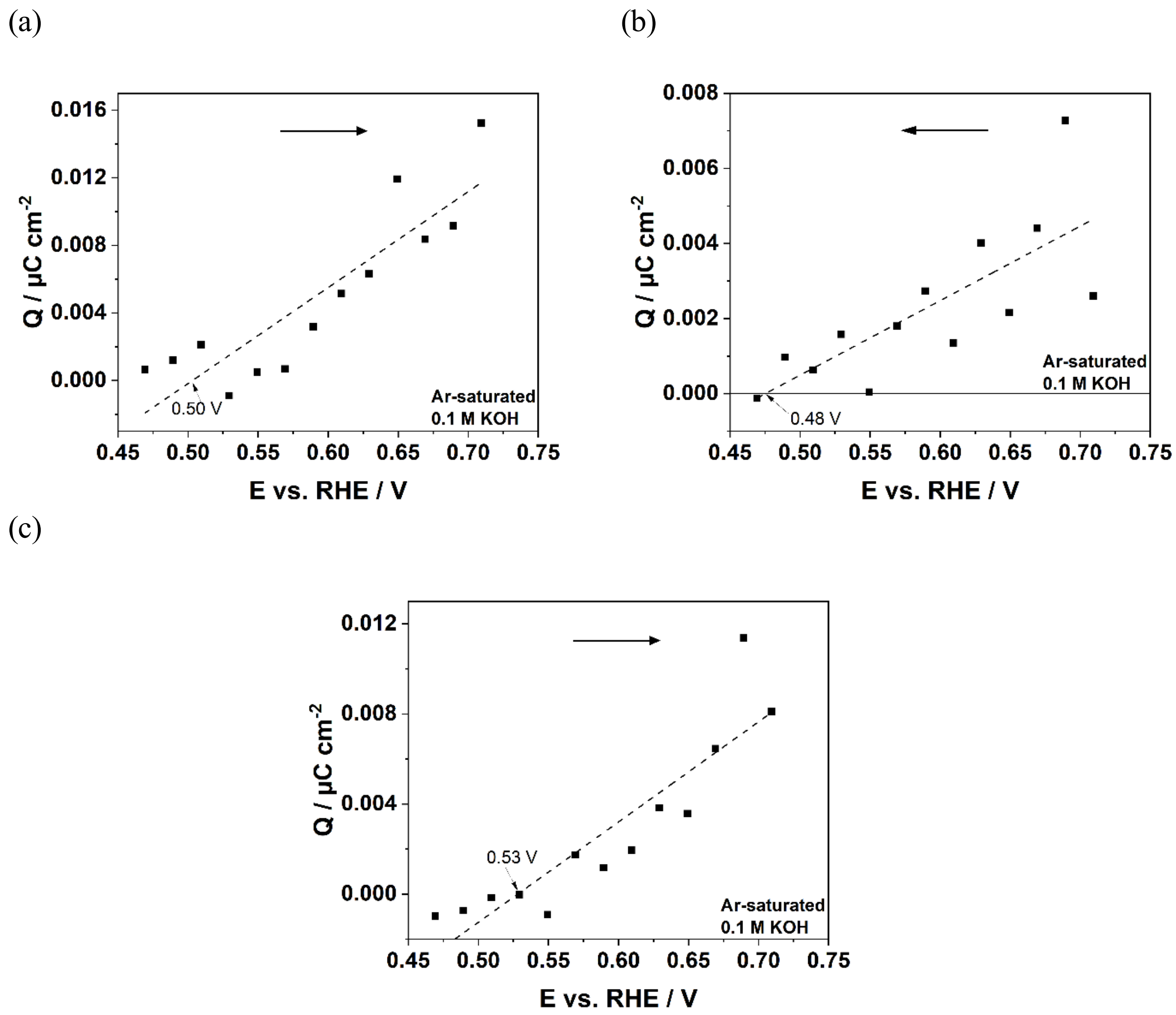

Figure S18. Surface charge as a function of the applied electrode potential for the Pd_{ML}/Au(111) working electrode in 0.1 M KOH. The transition point between negative and positive surface charge corresponds to the PME. The respective black arrows in (a-c) display the direction of the potential sweep in the experiment.

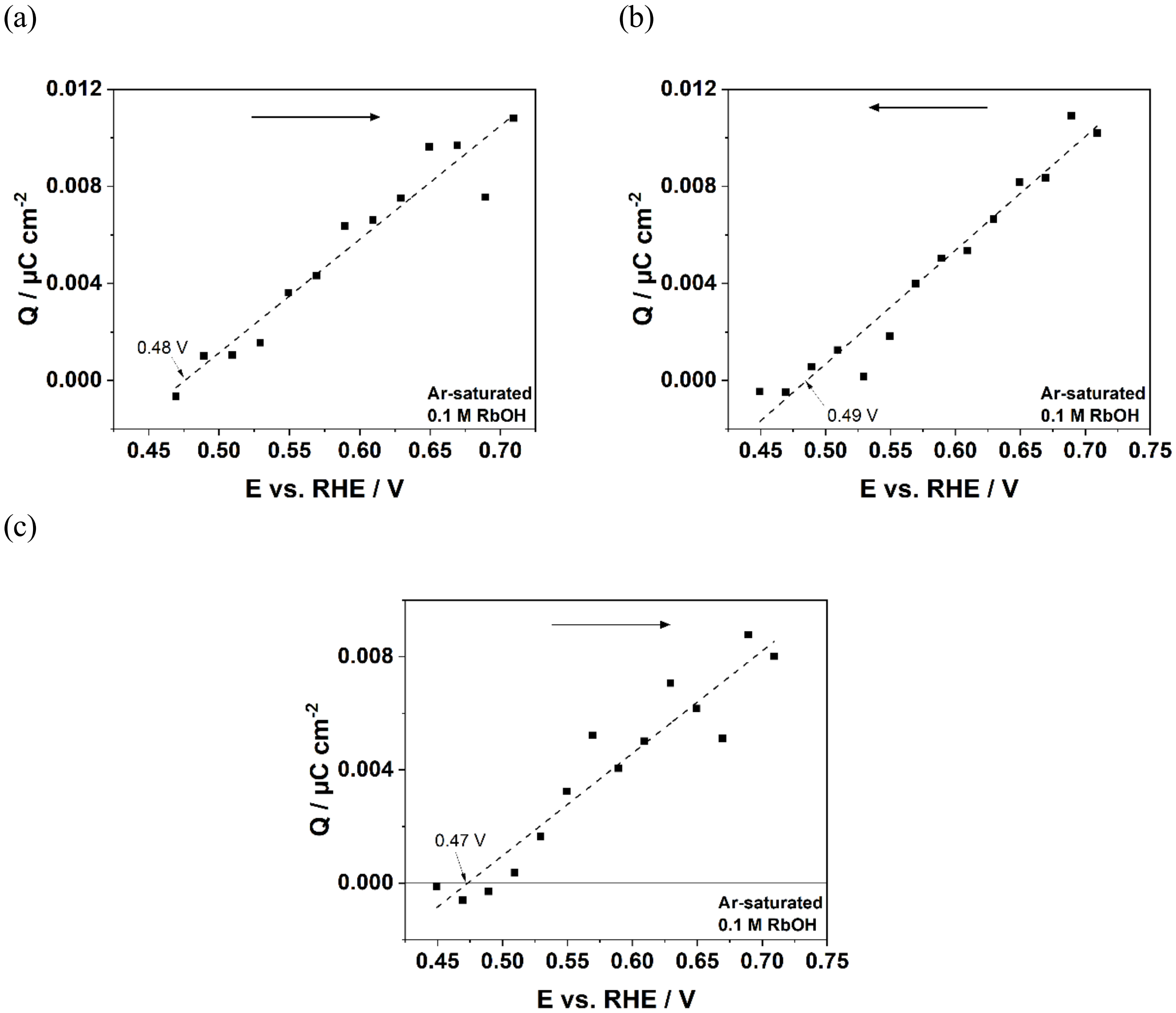

Figure S19. Surface charge as a function of the applied electrode potential for the Pd_{ML}/Au(111) working electrode in 0.1 M RbOH. The transition point between negative and positive surface charge corresponds to the PME. The respective black arrows in (a-c) display the direction of the potential sweep in the experiment.

(a)

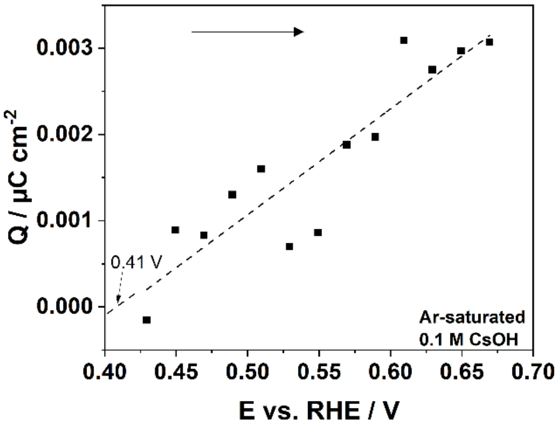

(b)

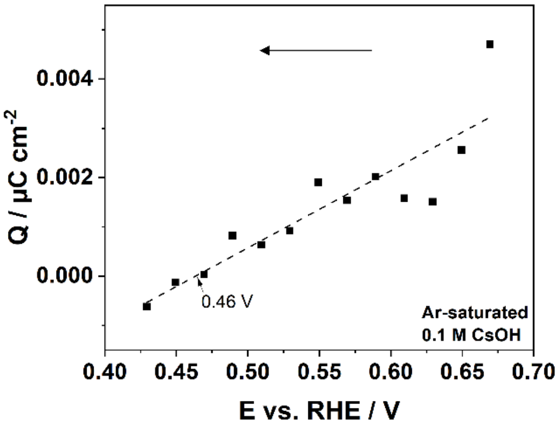

(c)

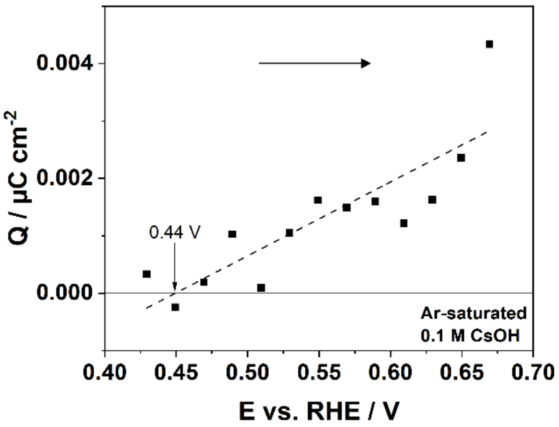

Figure S20. Surface charge as a function of the applied electrode potential for the Pd_{ML}/Au(111) working electrode in 0.1 M CsOH. The transition point between negative and positive surface charge corresponds to the PME. The respective black arrows in (a-c) display the direction of the potential sweep in the experiment.

---

[1] Mahmood, N.; Yao, Y.; Zhang, J.-W.; Pan, L.; Zhang, X.; Zou, J.-J. Electrocatalysts for Hydrogen Evolution in Alkaline Electrolytes: Mechanisms, Challenges, and Prospective Solutions. *Adv. Sci.* **2018**, 5(2), 1700464. DOI: 10.1002/advs.201700464.

[2] Bender, J. T.; Petersen, A. S.; Østergaard, F. C.; Wood, M. A.; Heffernan, S. M. J.; Milliron, D. J.; Rossmeisl, J.; Resasco, J. Understanding Cation Effects on the Hydrogen Evolution Reaction. *ACS Energy Lett.* **2023**, 8 (1), 657–665. DOI: 10.1021/acsenergylett.2c02500.
